# Microbiome Analysis of New, Insidious Cave Wall Alterations in the Apse of Lascaux Cave

**DOI:** 10.3390/microorganisms10122449

**Published:** 2022-12-12

**Authors:** Lise Alonso, Thomas Pommier, Danis Abrouk, Mylène Hugoni, Van Tran Van, Guillaume Minard, Claire Valiente Moro, Yvan Moënne-Loccoz

**Affiliations:** 1 Univ Lyon, Université Claude Bernard Lyon 1, CNRS, INRAe, VetAgro Sup, UMR5557 Ecologie Microbienne, F-69622 Villeurbanne, France; 2 Univ Lyon, INSA Lyon, CNRS, UMR5240 Microbiologie Adaptation et Pathogénie, F-69621 Villeurbanne, France; 3 Institut Universitaire de France (IUF), F-75005 Paris, France

**Keywords:** Paleolithic caves, anthropization, cave alterations, microbial diversity, pigmented fungi, collembola

## Abstract

Lascaux Cave is a UNESCO site that was closed to the public following wall surface alterations. Most black stains that had formed on wall surface are stable or receding, but a new type of alteration visually quite different (termed dark zones) developed in Lascaux’s Apse room in the last 15 years. Here, we tested the hypothesis that dark zones displayed a different microbial community than black stains previously documented in the same room, using metabarcoding (MiSeq sequencing). Indeed, dark zones, black stains and neighboring unstained parts displayed distinct microbial communities. However, similarly to what was observed in black stains, pigmented fungi such as *Ochroconis* (now *Scolecobasidium*) were more abundant and the bacteria *Pseudomonas* less abundant in dark zones than in unstained parts. The collembola *Folsomia candida*, which can disseminate microorganisms involved in black stain development, was also present on dark zones. Illumina sequencing evidenced *Ochroconis* (*Scolecobasidium*) in all collembola samples from dark zones, as in collembola from black stains. This study shows that the microbial properties of dark zones are peculiar, yet dark zones display a number of microbial resemblances with black stains, which suggests a possible role of collembola in promoting these two types of microbial alterations on wall surfaces.

## 1. Introduction

The calcareous region of Périgord in south-western France displays a wide range of karstic caves, and many of them are renowned for their exceptional parietal carvings, drawings and paintings [1,2]. Some of these caves can be visited by the general public, but tourism-related anthropization can lead to significant changes in microbial and arthropod diversity in comparison with pristine caves [3]. In most of these caves, tourism did not threaten cave wall quality and Paleolithic features. However, in Lascaux Cave, a stronger anthropization resulted in various cave wall alterations leading the authorities to close the cave to the public in 1963.

Cave wall alterations in Lascaux Cave included green stains (with the algae *Bracteacoccus minor*, in the 1950s) and white stains (due to the fungus *Fusarium solani*, in 2001), which were treated mechanically and with chemicals such as formaldehyde (to control algae), antibiotics (to control bacteria and fungi) and benzalkonium chloride (to control fungi) [2,4]. Starting at the end of 2001, and especially from 2006 on, stains that developed on cave walls were black, and black fungi such as *Ochroconis lascauxensis* (now reclassified as *Scolecobasidium lascauxense* [5,6]) have been evidenced in these stains [7]. The current management scheme in Lascaux Cave aims at minimizing human intervention. Most wall surface alterations have been stable in recent years and natural attenuation was even observed for some of them. Within Lascaux, however, the Apse remains a monitoring priority as this room still undergoes alteration development.

In Lascaux’s Apse, black stains visually resembling those present at other locations within the cave and thought to result from fungal synthesis of melanin [4,8,9] have been characterized microbiologically. These black stains occurring in lower parts of the Apse walls (below the ornate parts) are colonized by *Folsomia candida* collembola and display different microbial communities in comparison with unstained wall samples taken nearby [10]. It is thought that *F. candida* plays a key role in stain microbial dynamics by disseminating black fungi and feeding them through their feces [4,10].

**Figure 1:**
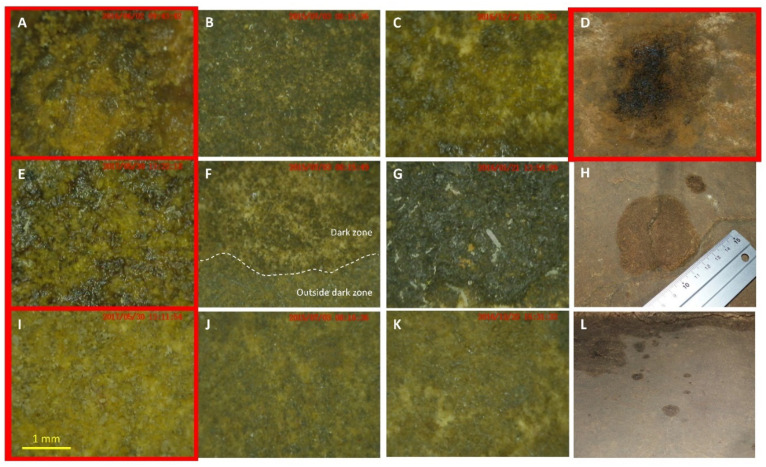
Photographs of dark zones (**H**,**L**) from the Apse compared with a black stain (**D**), and Dino-Lite microscope photographs of dark zones (**B**,**C**,**F**,**G**) compared with outside parts near dark zones (**J**,**K**), as well as black stains (**A**,**E**) and outside parts near a black stain (**I**). The reference situation of Apse black stains is shown in red frames, (**B**). (**F**) (showing the limit of a dark zone) and (**J**) correspond to left wall photographs, and (**C**). (**G**) (showing *F. candida* collembola) and (**K**) to right wall photographs.

The Apse walls also display small (centimetric) alterations that formed more recently than the black stains, which are not black while being quite distinct visually from the rest of the wall, and that have been termed dark zones by Lascaux monitoring staff. They are reminiscent of humid limestone surfaces, yet most of them occur on dry cave walls, distant from the few stratification joints where occasional signs of water film can be evidenced. They are rather insidious, as they remained inconspicuous until 2013, but afterwards the first dark zones were retrospectively identified on photographs taken back in 2008. Based on clearly distinct visual properties of black stains and dark zones (Figure 1), the current paradigm in Lascaux’s scientific board is to consider both phenomena as separate alteration processes. Whether these intriguing dark zones could represent a novel type of initial stage in the formation of black stains is not obvious, all the more as from 2001 to 2006, black stains formed directly, without going through a dark zone stage first. Therefore, longer-term monitoring of dark zones will be useful to answer this question. As in the case of black stains, they are colonized by collembola, and the occurrence of these dark zones in the same room of the cave raises questions on their origin, formation and microbiological properties.

The objective of this investigation was to characterize the microbial community of dark zones developing in the Apse of Lascaux, in comparison with healthy surfaces nearby. In addition, we aimed at determining microbiota specificities of dark zones vs. black stains, with the hypothesis that their microbial communities should differ since they are different alterations. Finally, we determined whether microorganisms associated to dark zones were also present in collembola present on the latter, to gain indirect insight into their dissemination potential via these arthropods.

## 2. Materials and Methods

### 2.1. Sampling

Sampling in Lascaux Cave (near Montignac, South-West France) was carried out mainly in the Apse, and was completed with a few samples from adjacent Nave room in February 2017. Sampling followed rules and regulation implemented to protect the cave, including the distribution of sampling activities over several days to limit human presence duration on a given day and wall samples taken by qualified restoration personnel. Five sampling campaigns were performed in late June–early July 2015, January 2016, June 2016, December 2016 and (for additional collembola) May 2017, using several areas selected on the left and right walls of the Apse.

Collembola and dark zones underneath were sampled for metabarcoding assessment of their associated microorganisms. A total of 17 dark zone samples of collembola (with 1 to 42 individuals per sample, for an average of 12 individuals, making 201 individuals in total) were obtained by sucking using sterile insect mouth aspirators (Rose Entomology, Benson, AZ, USA), whereas dark zones underneath and equivalent unstained, control areas located about 10 cm away were sampled using sterile swabs (3–6 samples per wall surface condition at each sampling date). All samples were placed into liquid nitrogen and transferred at −80 °C once in the lab prior to DNA extraction.

### 2.2. DNA Extraction, Identification of Collembola and Illumina Sequencing

DNA extractions from collembola were carried out under a laminar hood to avoid contamination. The 17 collembola samples (whole bodies were studied) were crushed using 1 mm beads in ATL lysis buffer (Qiagen, Hilden, Germany) that contained 20 mg·mL^−1^ lysozyme (Euromedex, Strasbourg, France), following homogenization 10 s in a Mini-beadbeaterTM (BioSpec Products, Bartlesville, OK, USA). After 2 h at 37 °C, 20 µL of proteinase K (20 mg·mL^−1^; Qiagen, Hilden, Germany) was added and samples were maintained 4 h with agitation (300 rpm) at 56 °C to achieve collembola lysis. DNA was extracted using Qiagen DNeasy Blood and Tissue kit (Qiagen, Hilden, Germany), following the manufacturer’s recommendations for both Gram-negative and Gram-positive bacteria, and elution was conducted using 12 µL. Final DNA concentration was measured using Nanodrop Safas UV-mc^2^ (SAFAS, Monaco, Principauté de Monaco), and DNA were kept at −20 °C.

DNA extraction from cave wall samples was performed using the FastDNA SPIN Kit for Soil (MP Biomedicals, Illkirch, France), following the manufacturer’s instructions. Elution was performed using two 50 µL volumes that were later combined, and final DNA concentration was measured using the Qubit dsDNA BR Assay Kit (Thermo Fisher Scientific, Eugene, OR, USA) according to manufacturer’s instructions. DNA extracts were kept at −20 °C.

Taxonomic identification of collembola was performed on four collembola samples, based on Sanger sequencing of a 708-bp fragment flanking gene *cox1*, after PCR amplification using 50 ng of DNA matrix and primers LCO1490 33 and HCO2198 [11], as described [10]. Sequencing was performed by Biofidal company (Lyon, France) and sequences (Bioproject number PRJEB27522) were blasted against the NCBI nr-database [12].

DNA extracts from cave walls and collembola were used to amplify the V3–V4 region of bacterial 16S rRNA genes with primers 341F and 805R [13], the second fungal internal transcribed spacers (ITS2) with primers ITS3_KYO2 and ITS4 [14], and (for cave wall DNA extracts only) the eukaryotic 18S rRNA genes with primers 18S_0067a_deg and NSR399 [15]. Illumina sequencing was implemented by Fasteris company (Geneva, Switzerland) from 1 µg of DNA extract, based on MiSeq paired-end chemistry (2 × 300 bp), with the goal of 70,000 paired reads per sample.

### 2.3. Bioinformatic Treatment of Illumina Sequence Data

Paired-end reads were demultiplexed. The adaptors were removed, as well as all sequences whose primer-complementing regions displayed at least two mismatches with the primer sequences, using a proprietary Perl script from Fasteris company. The sequences were then merged using Fast Length Adjustment of Short reads (FLASh) [16] with a maximum of 10% mismatch in the overlapped region. Denoising was performed by removing all reads that did not display the expected 200–500 bp length or exhibited ambiguous base(s) (N). The sequences were dereplicated and clustered using SWARM [17], on the basis of a local clustering threshold level and an aggregation distance of 3 to identify operational taxonomic units (OTUs). The finer taxonomic level thus obtained was the genus or the species depending on the taxa. Chimeras were removed using VSEARCH [18], as well as singletons and low-abundance sequences so as to keep only OTUs representing at least 0.005% of all sequences [19]. Taxonomic OTU affiliation at phylum, class, or genus/species level (with a focus on genera) was performed using RDP Classifier [20] against (i) the 119 SILVA database [21] for bacteria, (ii) the 123 SILVA database for micro-eukaryotes, and (iii) the UNITE database for fungal ITS2 [22]. The procedure was implemented in the FROGS pipeline [23]. The sequencing datasets containing merged paired-end reads have been deposited in EBI (reference PRJEB55380). For normalization, random resampling was carried out down to 6000 (bacteria), 26,674 (micro-eukaryotes) and 21,074 (fungi) sequences per sample, corresponding to the lowest sequence number per sample to enable comparisons between cave wall samples.

### 2.4. Statistical Analyses

The efficacy of sampling was evaluated using rarefaction curves on raw data (Figure S1). OTU richness was estimated with the Chao 1 index [24] while alpha-diversity, which combines both OTU richness and abundance, was estimated with both the Shannon’s H’ [25] and Simpson 1-D [26] indices, using Paleontological Statistics (PAST) software v3.14 [27]. Analysis of variance (ANOVA) followed with Tukey’s HSD tests were carried out to compare the number of OTUs or microbial diversity indices in/or outside dark zones (*p* < 0.05).

A comparison of the composition of the microbial communities was achieved using a non-metric multidimensional scaling (NMDS) based on Bray-Curtis dissimilarity matrix [28] after square-root transformation of data (to avoid over-dominance effects), using PAST v3.14. NMDS stress values below 0.1 are considered very good and those between 0.1 and 0.2 acceptable [29]. Then, analysis of variance using distance matrices (adonis) was performed, using the ‘vegan’ package in R (http://cran.r-project.org/web/packages/vegan/index.html, accessed on 28 February 2018), to assess significant differences (*p* < 0.05) in overall microbial community composition at the phylum or class levels. Pearson’s Chi-squared tests in R were used to investigate the proportions of phyla and genera in different microbial communities (*p* < 0.05).

## 3. Results

### 3.1. Genetic Structure of Fungal Community in Dark Zones

**Figure 2:**
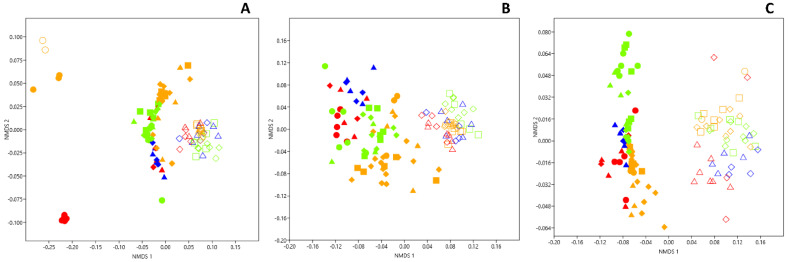
NMDS comparison of black stains (left wall samples in red and right wall samples in blue), dark zones (left wall samples in green and right wall samples in orange) and nearby unstained parts (empty symbols) from the left and right walls of Lascaux’s Apse taken in June–July 2015 (circles), January 2016 (squares), May–June 2016 (triangles) and December 2016 (diamonds), based on the relative proportion of both phyla and classes in the fungal (**A**), micro-eukaryotic (**B**) and bacterial communities (**C**). Data used for black stains and their neighboring unstained parts are those from Alonso et al. [10]. Stress values were 0.22, 0.19 and 0.17 for fungi, micro-eukaryotes and bacteria, respectively.

The hypothesis that dark zones could correspond to a different wall alteration process in comparison with black stains was assessed by Illumina sequencing (ITS2 genes), to determine whether or not the same fungal community was present in both. An NMDS comparison of Apse cave walls indicated that the structure of the fungal community differed clearly when comparing (i) dark zones with neighboring unstained parts (adonis F_1,68_ = 44.7, *p* = 0.001, R^2^ = 0.25), (ii) dark zones in June–July 2015 vs. January or December 2016 (adonis F_3,68_ = 7.99, *p* = 0.001, R^2^ = 0.06), and (iii) dark zones and black stains (adonis F_1,69_ = 15.13, *p* = 0.001, R^2^ = 0.11) (Figure 2A). Some differences were also found between (i) unstained parts next to dark zones vs. next to black stains (adonis F_3,38_ = 8.82, *p* = 0.001, R^2^ = 0.33), and (ii) dark zones from left vs. right walls (but not for unstained parts nearby) (adonis F_1,68_ = 11.6, *p* = 0.001, R^2^ = 0.06). The NMDS analysis carried out at the scale of the whole micro-eukaryotic community (i.e., 18S rRNA genes) confirmed the findings made with the fungal sub-compartment, but with (i) a clearer distinction between dark zones from left or right walls and (ii) less marked differences between sampling dates (Figure 2B).

### 3.2. Fungal Community Composition in Dark Zones

**Figure 3:**
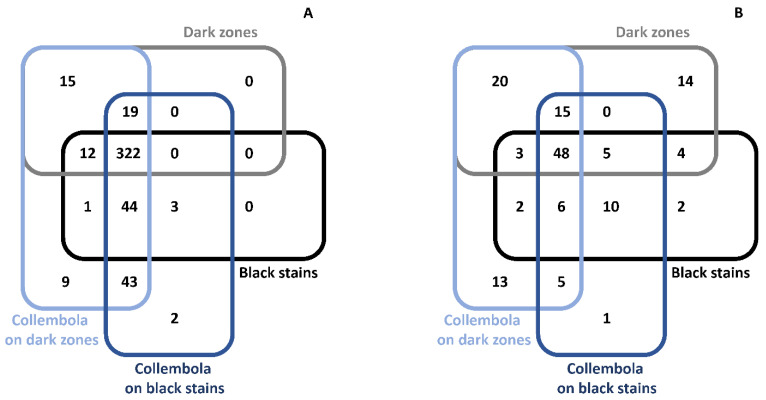
Venn diagrams showing unique and shared bacterial OTUs (**A**) and fungal OTUs (**B**) in collembola from dark zones, collembola from black stains, dark zones and black stains. Data used for black stains and black stain collembola are those from Alonso et al. [10].

**Figure 4:**
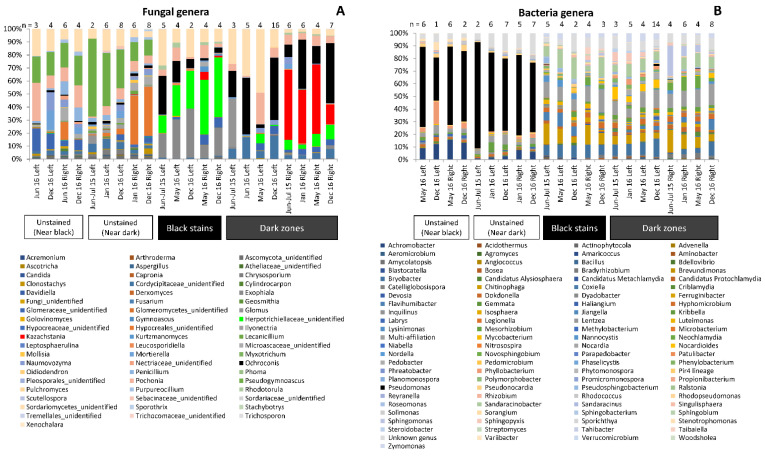
Community composition at genus level for fungi (**A**) and bacteria (**B**) in dark zones (Dark), black stains (Black) and nearby unstained parts (respectively Near Dark and Near Black) sampled from the left (L) and right (R) walls of Lascaux’s Apse in June–July 2015 (15J), January 2016 (16J), May–June 2016 (16M) and December 2016 (16D). Genera representing more than 1% of sequences are indicated. Each histogram is the average from 1–16 samples (indicated in each case). Data used for black stains and their neighboring unstained parts are those from Alonso et al. [10]. Detailed data are reported at Tables S1 and S2.

In Lascaux Cave, the development of black stains is attributed to melanin-producing fungi [4,9]. When considering OTUs, 60 of 109 fungal OTUs found in dark zones (i.e., 55%) were also present in black stains, which displayed only 20 (i.e., 25%) specific fungal OTUs (Figure 3B). The occurrence of black fungi in dark zones was investigated based on the taxonomic profile in fungal genera. First, the genus taxonomic profile of fungi in dark zones strongly differed from the one found in neighboring unstained parts (Chi-squared test, *p* < 0.0001), with noticeably *Pseudogymnoascus* less present in dark zones (Figure 4A, Table S1). For these dark zones, the genus taxonomic profile also differed (i) between left vs. right wall samples (*p* < 0.0001) (e.g., *Kazachstania* more prevalent on the right wall), and to a somewhat lesser extent (ii) between sampling times. Second, the genus taxonomic profile of dark zones was clearly distinct from the one of black stains, and for instance an unidentified *Cordycipitaceae* genus and *Kazachstania* were more prevalent in dark zones (Figure 4A). Third, as for black stains, the prevalence of pigmented fungal taxa was much higher in dark zones than in neighboring unstained parts, i.e., *Scolecobasidium* represented 37.6–47.8% of fungal sequences in dark zones in winter, 2.6–20.4% in dark zones in summer, vs. only 0.7–2.1% in neighboring unstained parts in winter or summer (Figure S2). In addition, fungal groups containing both pigmented and non-pigmented species/strains (i.e., in *Exophiala* and in Herpotrichiellaceae) amounted to 0.6–13.6% of fungal sequences in dark zones vs. only 0.05–0.92% in unstained parts (Figure S2). These trends were also observed when considering the genus taxonomic profile at the scale of the entire micro-eukaryotic community (Figure S3).

### 3.3. Fungal Community Size and Diversity in Dark Zones

**Figure 5:**
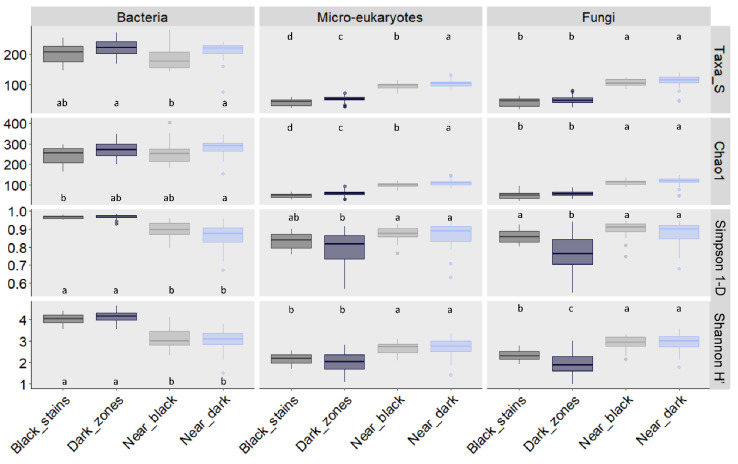
Number of taxa, Chao1 index of OTU richness, Simpson’s index of diversity and Shannon index of OTU diversity for bacteria, micro-eukaryotes and fungi in dark zones (Dark), black stains (Black) and nearby unstained parts (Near Dark and Near Black, respectively) sampled in Lascaux’s Apse walls. Data were combined for the left and right walls sampled in June–July 2015, January 2016, May–June 2016 and December 2016. The differences between conditions are shown with lowercase letters (based on ANOVA and Tukey’s tests; *p* < 0.05). Data used for black stains and their neighboring unstained parts are those from Alonso et al. [10].

Whether taxonomic findings made with fungi in dark zones could result from a bias linked to lower amounts of microorganisms there was explored by quantitative PCR of 18S rRNA genes. Similar microbial densities of microeukaryotes were evidenced for unstained parts and dark zones (Figure S4B). Since some of the fungi increased in relative abundance in dark zones, it resulted in lower fungal diversity in dark zones than in unstained parts, as shown by smaller number of taxa, Chao1 richness index, Simpson index and Shannon index (Figure 5), which were at comparable levels as those in black stains [10]. These fungal diversity indices for dark zones did not differ significantly when comparing sampling times (Figure S5) or right and left walls (Figure S6).

### 3.4. Genetic Structure of Bacterial Community in Dark Zones

NMDS comparison of Apse cave walls indicated that the structure of the bacterial community differed when comparing (i) dark zones with neighboring unstained parts (adonis F_1,69_ = 115.5, *p* = 0.001, R^2^ = 0.49) and (ii) dark zones with black stains (adonis F_1,69_ = 8.69, *p* = 0.001, R^2^ = 0.08). It differed also when comparing (i) unstained parts next to dark zones vs. unstained parts next to black stains (adonis F_1,41_ = 9.78, *p* = 0.001, R^2^ = 0.13), (ii) left vs. right walls (adonis F_1,69_ = 13.58, *p* = 0.001, R^2^ = 0.06) for dark zones (but not for unstained parts), and (iii) different samplings (adonis F_3,69_ = 5.62, *p* = 0.001, R^2^ = 0.07) (Figure 2C).

### 3.5. Bacterial Community Composition in Dark Zones

When considering OTUs, 334 of 368 bacterial OTUs found in dark zones (i.e., 91%) were also present in black stains, which displayed only 48 (i.e., 13%) specific bacterial OTUs (Figure 3A). The proportion of unaffiliated sequences was 20% in dark zones and black stains, 16% in unstained parts near dark zones and 8% in unstained parts near black stains. In unstained parts of the Apse, the genus taxonomic profile of bacteria was rather comparable in all samples, with minor differences for samples taken close to dark zones vs. close to black stains. In contrast, bacterial community composition was very different in dark zones vs. in neighboring unstained parts (Chi-squared test, *p* < 0.0001). A key feature of the bacterial community in black stains was the very low amount of *Pseudomonas*, which predominated in unstained parts [10], and levels of *Pseudomonas* were low in dark zones also (0.01–1.6%). In addition, levels of *Neochlamydia* within dark zones were higher in right walls than left walls (2.5–11% vs. 0.01–2.5%), and minor differences were found between different dark zones from a same wall or different sampling dates for a same dark zone (Figure 4B, Table S2).

### 3.6. Bacterial Community Size and Diversity in Dark Zones

As for fungi, the findings made with bacteria did not coincide with biologically-lower bacterial numbers in dark zones, as indicated by quantitative PCR data of 16S rRNA genes (Figure S4A). As in black stains, the much lower prevalence of *Pseudomonas* in dark zones than in unstained parts enabled proliferation of many other bacterial taxa, as indicated by higher Simpson and Shannon indices in comparison with these unstained parts (Figure 5).

### 3.7. Microbial Comparison of Dark Zones in the Apse and the Nave

**Figure 6:**
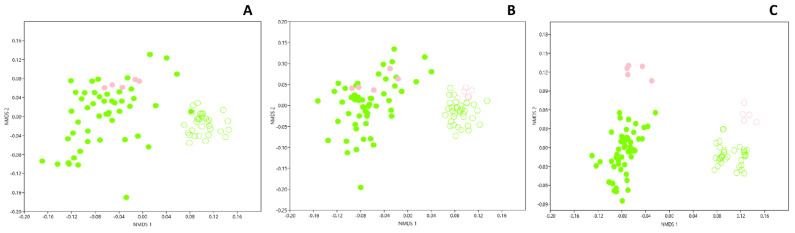
NMDS comparison of dark zones and nearby unstained parts from the walls of Lascaux’s Apse taken in June–July 2015, January 2016, May–June 2016, December 2016 and from the Nave in February 2017, based on the relative proportion of both phyla and classes in the fungal (**A**), micro-eukaryotic (**B**) and bacterial communities (**C**). Apse data for dark zones and their neighboring unstained parts are those already shown in Figure 2 for the same sampling dates. Stress values were 0.19, 0.19 and 0.15 for fungi, micro-eukaryotes and bacteria, respectively. Green circles represent Apse samples, pink circles Nave samples, and empty symbols unstained parts of the Apse and Nave.

A few dark zones have also been evidenced on walls of the Nave, a room contiguous but perpendicular to the Apse, and they were sampled in February 2017 and processed as for Apse samples. NMDS of ITS2 data showed that the fungal community in dark zones did not differ when comparing Apse and Nave samples, whereas the comparison was not possible for neighboring unstained parts as ITS2 sequencing for these samples was not successful (Figure 6A).

When considering the micro-eukaryotic community more globally (i.e., 18S rRNA genes), NMDS indicated that dark zones did not differ in Apse and Nave, whereas unstained parts in Apse and Nave did differ (Figure 6B). Importantly, the micro-eukaryotic community was not the same in dark zones vs. neighboring unstained parts, both in the Apse and the Nave.

When considering the bacterial community, NMDS indicated that dark zones differed from unstained parts in the Nave, and differences (of lower magnitude) were also found between Nave and Apse samples, regardless of whether dark zones or unstained parts were studied (Figure 6C).

### 3.8. Microbiota Associated with Collembola Present on Dark Zones

Collembola have been identified as potential disseminators of bacteria and fungi located in black stains [10,30], and black stain taxa were also identified in collembola [10]. Whether a similar collembola-associated microbiota occurs in collembola sampled from dark zones was investigated by MiSeq sequencing.

A total of 112 ITS2-based fungal OTUs were found in collembola from dark zones, including the genera *Acremonium*, *Exophiala*, *Scolecobasidium* (corresponding to *S. lascauxense* based on ITS2 analysis), all with black pigmentation potential, and *Alternaria,* of uncertain pigmentation potential. In addition, 66 of the 109 fungal OTUs found in dark zones (i.e., 61%) were also evidenced in collembola sampled on these dark zones (Figure 3B). Collembola from dark zones versus black stains shared 74 fungal OTUs, which represented 66% of the 112 OTUs found with dark-zone collembola (and 82% of the 90 OTUs found with black-stain collembola).

A total of 465 bacterial OTUs were documented in collembola from dark zones, based on 16S rRNA gene data, including the endosymbiont *Wolbachia* (representing 3.5% of all bacterial sequences). All 368 bacterial OTUs found in the dark zones underneath were also part of the 465 OTUs present in collembola (Figure 3A). In addition, collembola from dark zones versus from black stains shared 428 bacterial OTUs, which represented 92% of the 465 OTUs found with dark zone collembola (and 99% of the 433 OTUs found with black stain collembola).

## 4. Discussion

Dark zones and black stains represent major wall surface alterations in lower areas of the Apse. They are visually very different and show different formation dynamics (e.g., only dark zones keep developing in the Apse), but both are largely colonized by collembola belonging to the same species *Folsomia candida*. Whether dark zones present the same microbial features as *bona fide* black stains was investigated by Illumina sequencing, which highlighted remarkable similarities between both, i.e., (i) microbial communities that differed clearly from neighboring unstained parts in both cases, (ii) the prevalence of *Scolecobasidium* and other pigmented fungi in contrast to the situation in unstained parts, (iii) the occurrence of these fungal taxa (and of numerous other microbial taxa colonizing altered surfaces) in *F. candida* sampled from the same dark zones or black stains, (iv) very sparse populations of *Pseudomonas* spp. even though these bacteria were predominant in unstained parts, (v) similar numbers of taxa, Chao1 richness indices, Simpson indices and Shannon indices, both for fungi and for bacteria, and (vi) similar quantitative PCR levels, both for 18S and for 16S rRNA genes. However, some microbial differences were also shown between dark zones and black stains, especially (i) in the structure of the fungal community and of the bacterial community (NMDS analysis), (ii) in OTU composition of surface alterations since 45% of fungal OTUs and 9% of bacterial OTUs found in dark zones were not present in black stains, and (iii) in OTU composition of collembola taken from surface alterations since 34% of fungal OTUs and 8% of bacterial OTUs found in dark-zone collembola were not present in black-stain collembola samples.

The occurrence, albeit at low levels, of bacterial taxa typically associated with amoebas, i.e., *Legionella*, *Nordella*, *Bosea*, *Neochlamydia*, *Candidatus* Protochlamydia, *Candidatus* Neochlamydia (Figure 4) points to the significance of amoebal hosts, as evidenced in Figure S3. However, non-fungal microeukaryotic taxa were found in low sequences numbers, which were similar inside and outside dark zones. The analysis of Archaea was also attempted but failed to produce sufficient Illumina sequences for analysis.

This study also showed that microbial communities differed when comparing unstained parts sampled next to dark zones and unstained parts close to black stains. On one hand, these differences could merely result from the spatial heterogeneity of microbial geographic distribution within Lascaux Cave, as suggested when comparing different areas on a same geological substrate in the Passage e.g., within the limestone inclined planes [31]. This hypothesis is strengthened by NMDS findings for unstained parts of the Nave, sampled on the same substrate and in contiguous locations in comparison to the Apse, which also showed differences with Apse unstained parts. On the other hand, the possibility exists that these differences could reflect variability in (micro)environmental conditions on cave wall surfaces, which might translate into different conditions of microbial colonization and functioning and lead to different types of alteration processes. This possibility is substantiated by the fact that dark zones and black stains do not occur exactly at the same vertical level of the walls in the Apse (Figure S7). Both scenarios are not mutually exclusive, which highlights the need to better understand the microbial and ecological mechanisms involved in wall surface alteration(s).

Whereas dark zones and black stains developed as separate visual alterations for years (since 2008 and 2001, respectively), different dynamics were noticed in recent years as black stains remained with the same visual appearance once they were formed, but certain dark zones evolved and a black stain (Figure S8) progressively formed in the middle (without leading there to distinct microbial communities, however; Figure S9). This may seem surprising, since (i) visual properties of dark zones and black stains (Figure 1) point to distinct formation processes, and (ii) all black stains monitored so far, in the Apse and elsewhere, formed without going through a preliminary dark-zone stage. However, this observation raises the possibility that a novel, multi-stage process of black stain formation had been started in the Apse, resulting perhaps from different priority effects in community assembly [32] which might, in turn, account for differences in early microbial functioning [33]. Further monitoring will be needed to determine whether all dark zones are bound to form a black stain at some point of time, or if this process is restricted to a few dark zones present in specific Apse locations.

An intriguing feature of both dark zones and black stains is the abundance of *F. candida*, whereas these collembola are very seldom found outside of wall surface alterations in the Apse. The ecological significance of collembola in Lascaux has already been investigated [4,7,30], including in the specific case of black stains formed in the Apse [10]. Results point to two roles played by collembola in relation to black stain formation. First, *F. candida* collembola could be involved in dissemination of particular microorganisms including *Scolecobasidium* spp. and other black fungi, based on their splattering on black stains [10,30], ingestion of black fungus biomass and presence of viable conidia in fecal pellets when studied in the laboratory [30], deposition of fecal pellets away from fungal colonies [30] and dissemination potential of black stain microorganisms themselves [10]. This possibility is likely for dark zones as well, since the same collembola species was found there, microbial taxa present on black stains and potentially disseminated by *F. candida* (especially black fungi) were also established in dark zones, and many of the latter were evidenced in collembola sampled on dark zones. Second, *F. candida* collembola are thought to be involved in recycling of microbial biomass and differential selection of microbial taxa, based on assimilation of microbial C and black stain constituents [10], decay of fungal colonies [30] and taxa-specific predatory trimming of bacteria when in high numbers [34] in vitro. Perhaps these collembola contribute to counter-selection of *Pseudomonas* bacteria, which might produce antifungal metabolites and limit the development of pigmented fungi on unmarked surfaces (as proposed before [10]). These *F. candida* effects are thought to promote establishment of black fungi [30] and subsequent melanin synthesis [30], but obviously this does not take place as such in dark zones, where the black color associated to melanin is not observed (at least as long as dark zones remain without any black-stain center). Instead, visual observations (Figure 1F) suggest modifications of the surface of unstained areas (possibly by consumption of surface constituents) rather than microbial build-up and melanin deposition. Therefore, further microscopy and transcriptomics-based assessments would be needed to decipher microbial processes in action.

In conclusion, this investigation showed that largely similar microbial dynamics were at play in dark zones compared with black stains of Lascaux’s Apse, with hypothetically the same role of collembola in promoting microbial dissemination and driving microbial selection. It also showed that very different visual properties could result from the functioning of microbial communities with seemingly-minor differences in structure and composition and colonizing similar (but not identical) types of limestone surfaces in Lascaux’s Apse. These differences in microbial functioning need to be targeted to better understand cave wall alterations in Lascaux Cave and especially the more recent, insidious dark zones.

## Data Availability

Sequence data are available at EBI (reference PRJEB55380).

## Supplementary Materials

The following supporting information can be downloaded at: https://www.mdpi.com/article/10.3390/microorganisms10122449/s1, supplementary data are available at microorganisms online.
