# Supplementary material for: Microbiome Analysis of New, Insidious Cave Wall Alterations in the Apse of Lascaux Cave
Source: Microorganisms. 2022 Dec 12;10(12):2449. doi: 10.3390/microorganisms10122449 (PMC9785961; doi:10.3390/microorganisms10122449)
Supplement: Supplementary file 1 [file microorganisms-10-02449-s001.zip › Abside_ZS_figures sup 031222.pptx]

## Slide 1
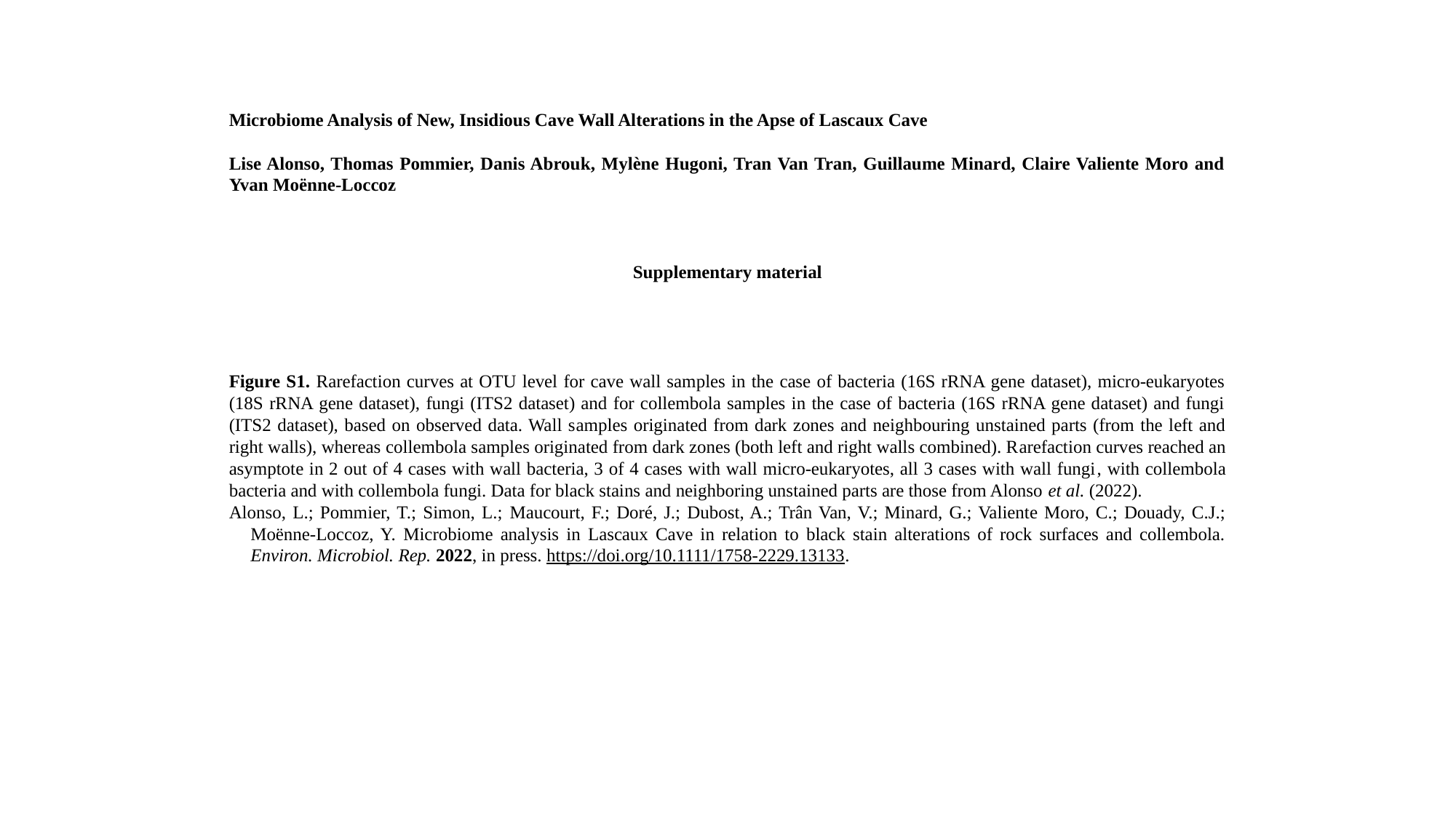

Microbiome Analysis of New, Insidious Cave Wall Alterations in the Apse of Lascaux Cave
Lise Alonso, Thomas Pommier, Danis Abrouk, Mylène Hugoni, Tran Van Tran, Guillaume Minard, Claire Valiente Moro and Yvan Moënne-Loccoz
Supplementary material
Figure S1. Rarefaction curves at OTU level for cave wall samples in the case of bacteria (16S rRNA gene dataset), micro-eukaryotes (18S rRNA gene dataset), fungi (ITS2 dataset) and for collembola samples in the case of bacteria (16S rRNA gene dataset) and fungi (ITS2 dataset), based on observed data. Wall samples originated from dark zones and neighbouring unstained parts (from the left and right walls), whereas collembola samples originated from dark zones (both left and right walls combined). Rarefaction curves reached an asymptote in 2 out of 4 cases with wall bacteria, 3 of 4 cases with wall micro-eukaryotes, all 3 cases with wall fungi, with collembola bacteria and with collembola fungi. Data for black stains and neighboring unstained parts are those from Alonso et al. (2022).
Alonso, L.; Pommier, T.; Simon, L.; Maucourt, F.; Doré, J.; Dubost, A.; Trân Van, V.; Minard, G.; Valiente Moro, C.; Douady, C.J.; Moënne-Loccoz, Y. Microbiome analysis in Lascaux Cave in relation to black stain alterations of rock surfaces and collembola. Environ. Microbiol. Rep. 2022, in press. https://doi.org/10.1111/1758-2229.13133.

## Slide 2
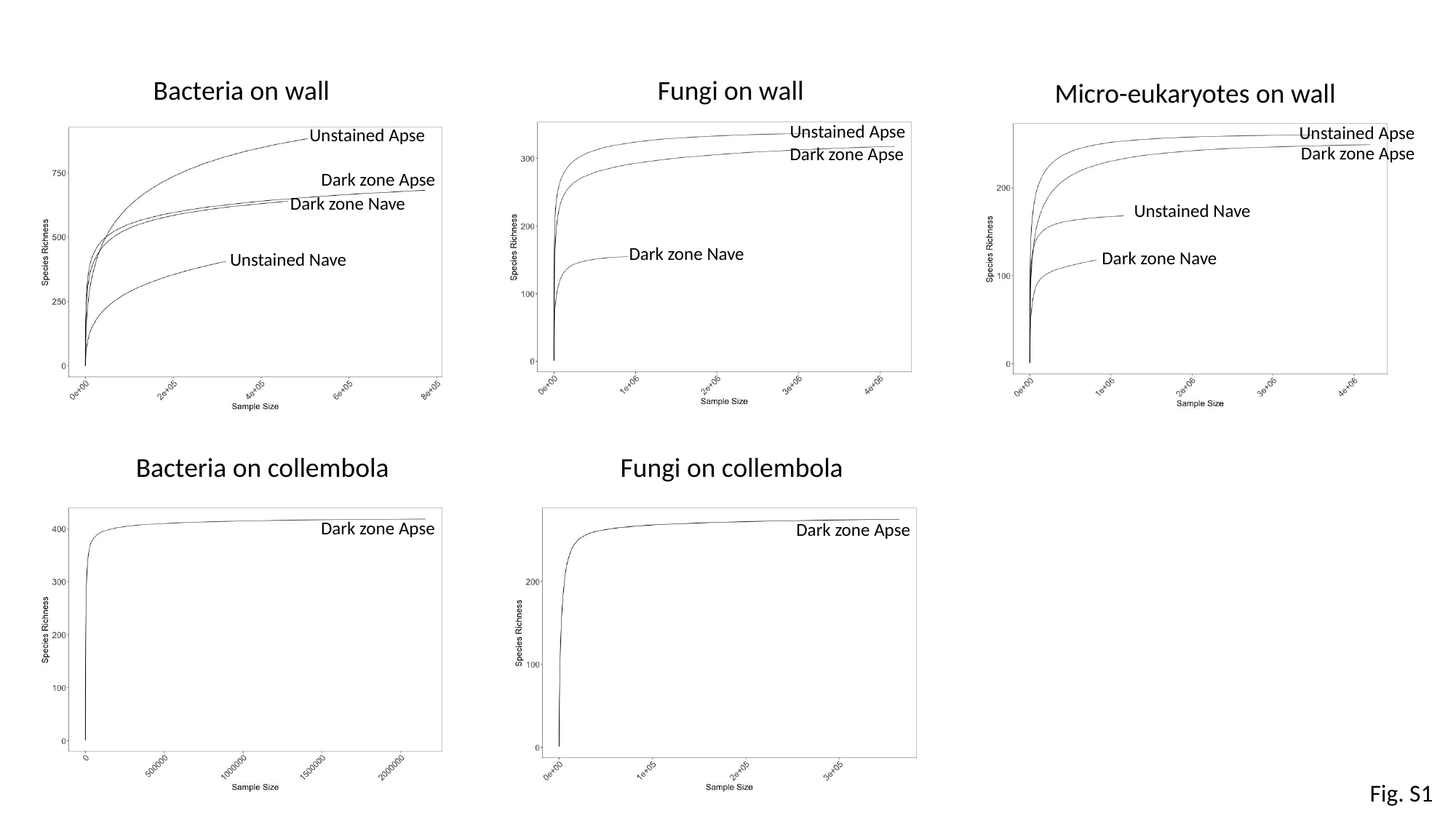

Bacteria on wall
Fungi on wall
Micro-eukaryotes on wall
Unstained Apse
Unstained Apse
Unstained Apse
Dark zone Apse
Dark zone Apse
Dark zone Apse
Dark zone Nave
Unstained Nave
Dark zone Nave
Dark zone Nave
Unstained Nave
Bacteria on collembola
Fungi on collembola
Dark zone Apse
Dark zone Apse
Fig. S1

## Slide 3
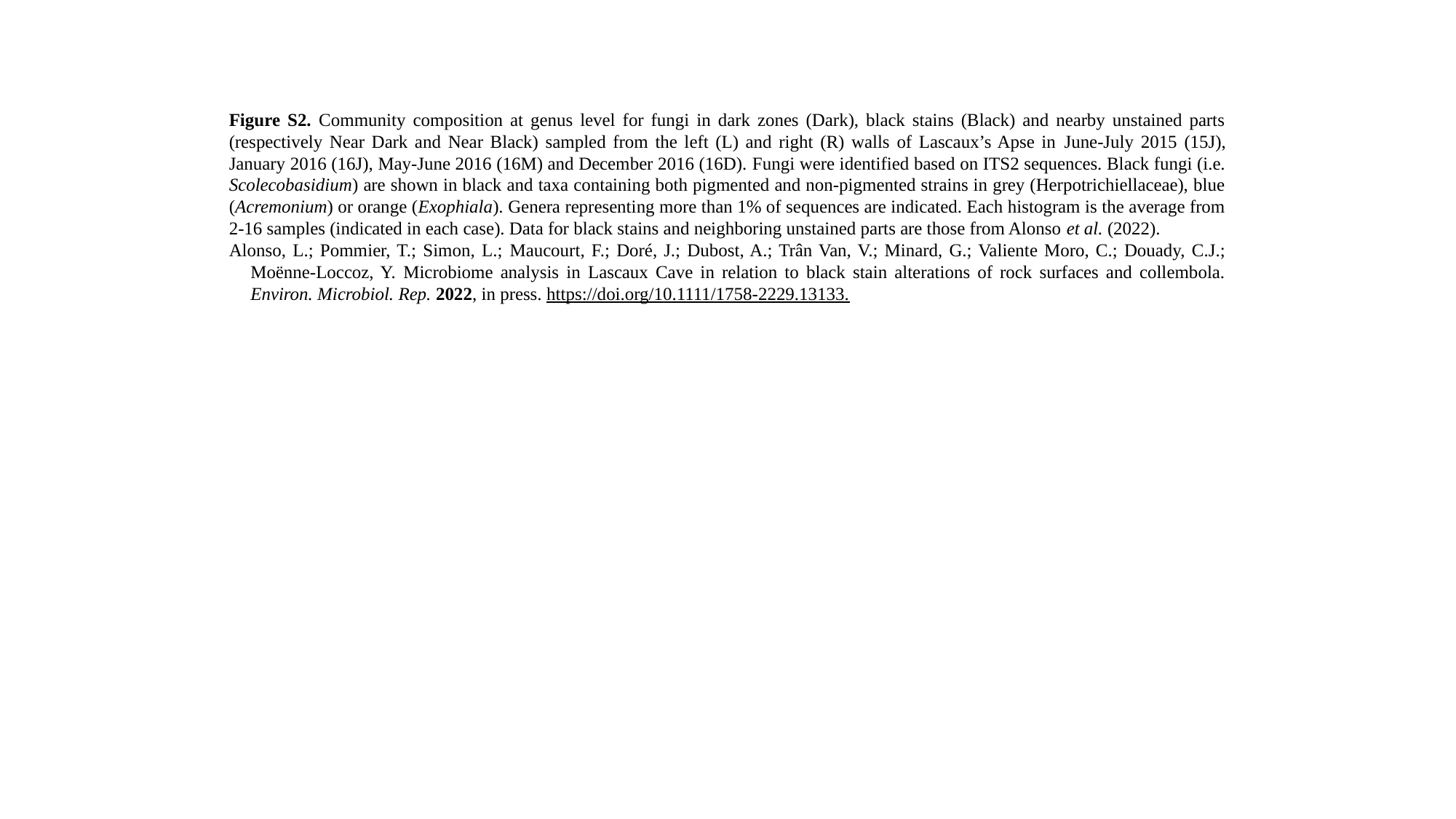

Figure S2. Community composition at genus level for fungi in dark zones (Dark), black stains (Black) and nearby unstained parts (respectively Near Dark and Near Black) sampled from the left (L) and right (R) walls of Lascaux’s Apse in June-July 2015 (15J), January 2016 (16J), May-June 2016 (16M) and December 2016 (16D). Fungi were identified based on ITS2 sequences. Black fungi (i.e. Scolecobasidium) are shown in black and taxa containing both pigmented and non-pigmented strains in grey (Herpotrichiellaceae), blue (Acremonium) or orange (Exophiala). Genera representing more than 1% of sequences are indicated. Each histogram is the average from 2-16 samples (indicated in each case). Data for black stains and neighboring unstained parts are those from Alonso et al. (2022).
Alonso, L.; Pommier, T.; Simon, L.; Maucourt, F.; Doré, J.; Dubost, A.; Trân Van, V.; Minard, G.; Valiente Moro, C.; Douady, C.J.; Moënne-Loccoz, Y. Microbiome analysis in Lascaux Cave in relation to black stain alterations of rock surfaces and collembola. Environ. Microbiol. Rep. 2022, in press. https://doi.org/10.1111/1758-2229.13133.

## Slide 4
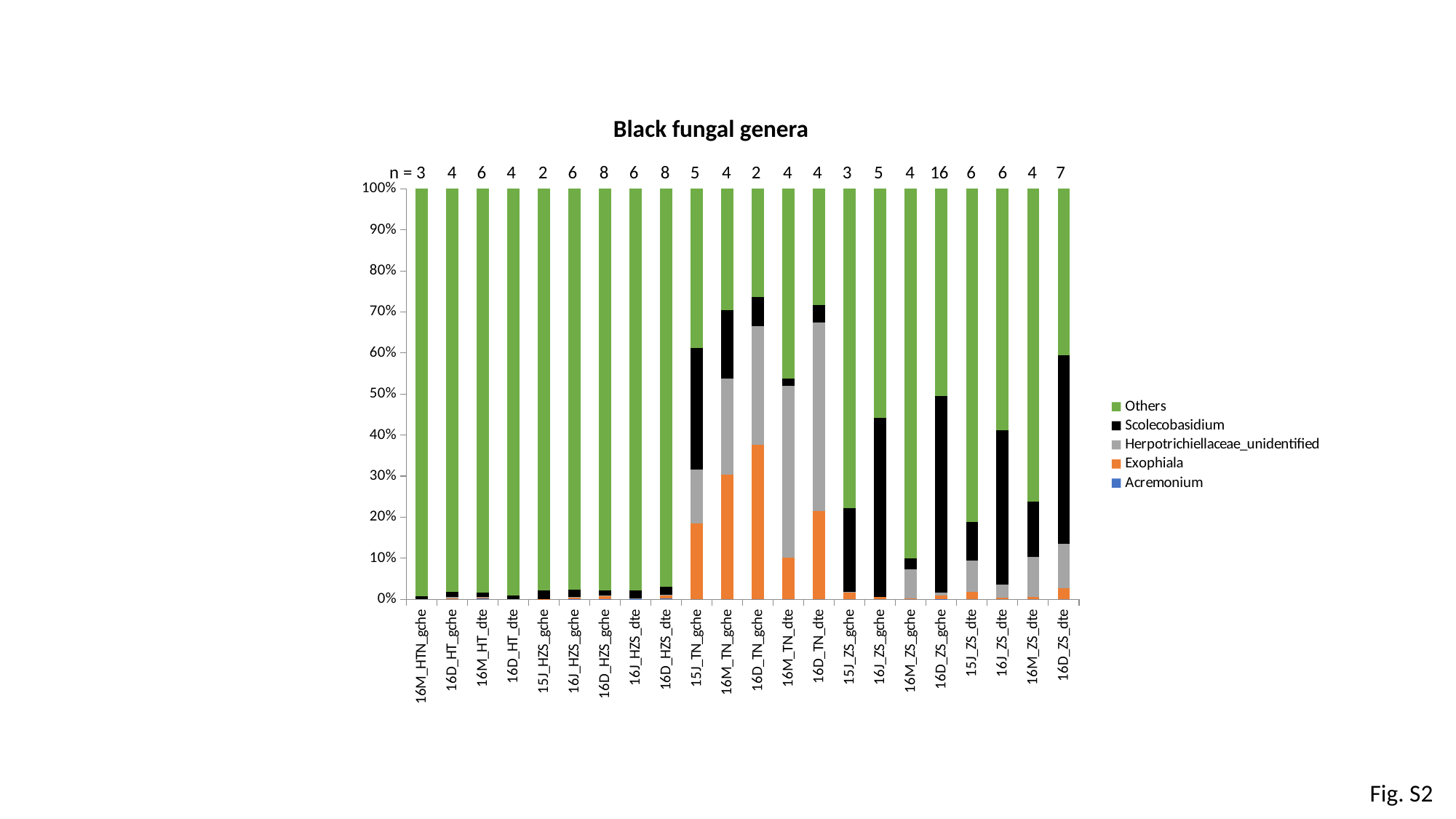

Black fungal genera
n = 3
4
6
4
2
6
8
6
8
5
4
2
4
4
3
5
4
16
6
6
4
7
### Chart
| Category | Acremonium | Exophiala | Herpotrichiellaceae_unidentified | Scolecobasidium | Others |
|---|---|---|---|---|---|
| 16M_HTN_gche | 0.0 | 0.05852393154281737 | 0.012653823036284838 | 0.6738160766821676 | 99.25500616873873 |
| 16D_HT_gche | 0.0 | 0.2574262123944197 | 0.25505362057511627 | 1.3713580715573694 | 98.11616209547309 |
| 16M_HT_dte | 0.2538673246654645 | 0.21511499161684225 | 0.01739900667489165 | 1.1222359305305116 | 98.39138274651229 |
| 16D_HT_dte | 0.0 | 0.0652462750308437 | 0.014235550915820442 | 0.7841415962797761 | 99.13637657777356 |
| 15J_HZS_gche | 0.0 | 0.05456961184397836 | 0.0 | 2.0949985764449086 | 97.85043181171112 |
| 16J_HZS_gche | 0.021353326373730663 | 0.3471892695580652 | 0.11704786308563474 | 1.852994210875961 | 97.6614153301066 |
| 16D_HZS_gche | 0.0005931479548258518 | 0.8677754579102211 | 0.059907943437411036 | 1.2853516181076208 | 97.78637183258992 |
| 16J_HZS_dte | 0.05852393154281737 | 0.10360317610958211 | 0.03242542153047989 | 1.924171965455063 | 97.88127550536205 |
| 16D_HZS_dte | 0.1465075448419854 | 0.7746512290025623 | 0.09490367277213628 | 2.060002847110183 | 96.92393470627313 |
| 15J_TN_gche | 0.0 | 18.44452880326469 | 13.131821201480498 | 29.618487235456016 | 38.805162759798804 |
| 16M_TN_gche | 0.0 | 30.316978267058936 | 23.528993072031888 | 16.502562399164848 | 29.651466261744332 |
| 16D_TN_gche | 0.0 | 37.63167884597134 | 28.895795767296196 | 7.0750688051627595 | 26.397456581569706 |
| 16M_TN_dte | 0.0 | 10.130967068425548 | 41.92251115118155 | 1.7367372117300939 | 46.20978456866281 |
| 16D_TN_dte | 0.0 | 21.466024485147575 | 45.99981019265446 | 4.153221979690614 | 28.380943342507354 |
| 15J_ZS_gche | 0.0 | 1.7272468444528801 | 0.004745183638606814 | 20.421688652684193 | 77.84631931922432 |
| 16J_ZS_gche | 0.0 | 0.6026383221030653 | 0.0018980734554427258 | 43.63196355698965 | 55.76350004745184 |
| 16M_ZS_gche | 0.0 | 0.18506216190566574 | 7.156923222928728 | 2.6205276644206132 | 90.03748695074499 |
| 16D_ZS_gche | 0.008007497390148999 | 1.0027166176331024 | 0.6844927398690329 | 47.822257283856885 | 50.48252586125083 |
| 15J_ZS_dte | 0.0 | 1.7675809053810383 | 7.7354401948688745 | 9.39625446838126 | 81.10072443136883 |
| 16J_ZS_dte | 0.0 | 0.4658188605232356 | 3.068552086299073 | 37.66094081174275 | 58.804688241434945 |
| 16M_ZS_dte | 0.0 | 0.5895890670968966 | 9.656448704564866 | 13.634098889627028 | 76.11986333871121 |
| 16D_ZS_dte | 0.0 | 2.6952643067286703 | 10.877994549817648 | 45.92592090456758 | 40.50082023888611 |Fig. S2

## Slide 5
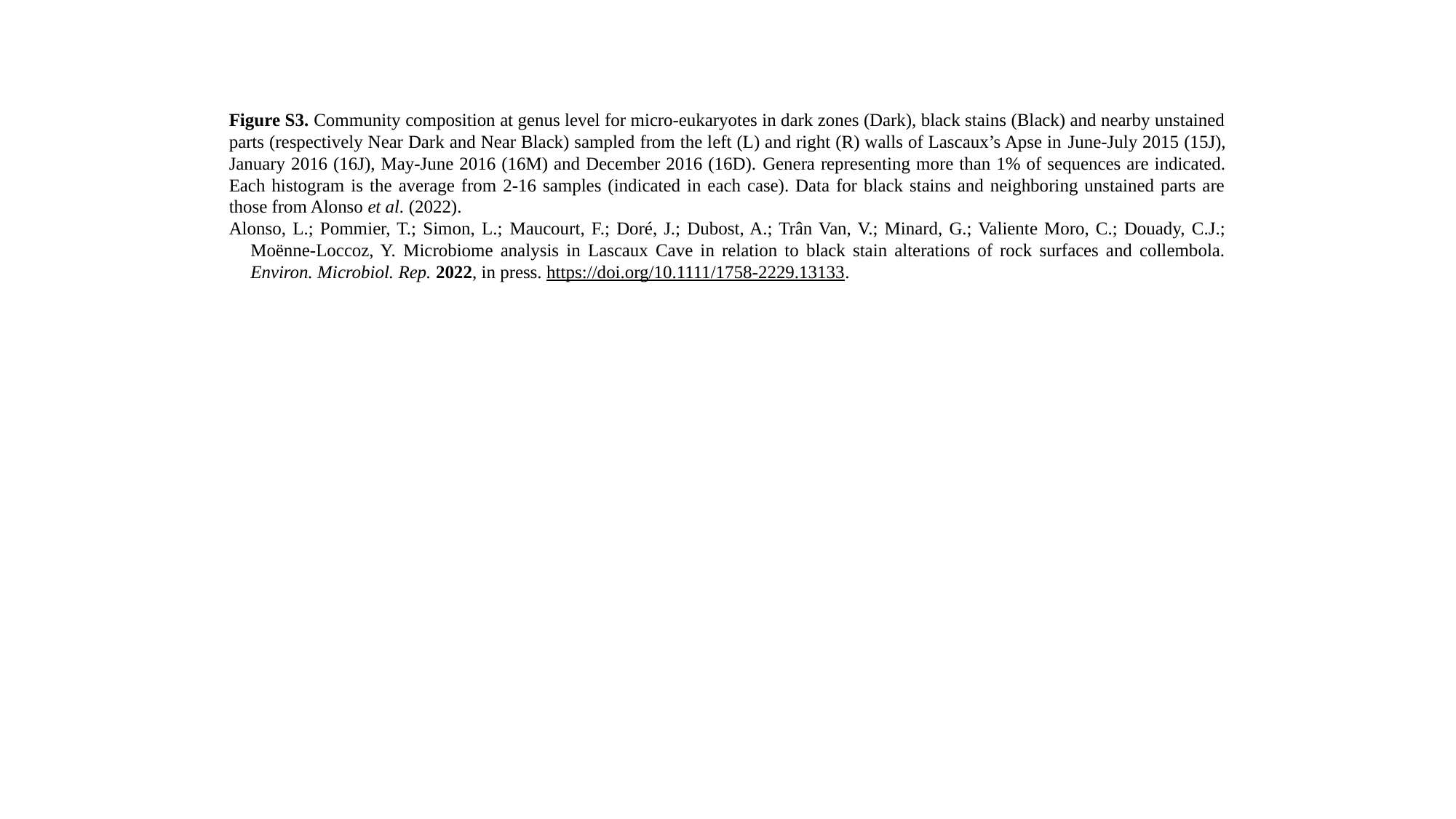

Figure S3. Community composition at genus level for micro-eukaryotes in dark zones (Dark), black stains (Black) and nearby unstained parts (respectively Near Dark and Near Black) sampled from the left (L) and right (R) walls of Lascaux’s Apse in June-July 2015 (15J), January 2016 (16J), May-June 2016 (16M) and December 2016 (16D). Genera representing more than 1% of sequences are indicated. Each histogram is the average from 2-16 samples (indicated in each case). Data for black stains and neighboring unstained parts are those from Alonso et al. (2022).
Alonso, L.; Pommier, T.; Simon, L.; Maucourt, F.; Doré, J.; Dubost, A.; Trân Van, V.; Minard, G.; Valiente Moro, C.; Douady, C.J.; Moënne-Loccoz, Y. Microbiome analysis in Lascaux Cave in relation to black stain alterations of rock surfaces and collembola. Environ. Microbiol. Rep. 2022, in press. https://doi.org/10.1111/1758-2229.13133.

## Slide 6
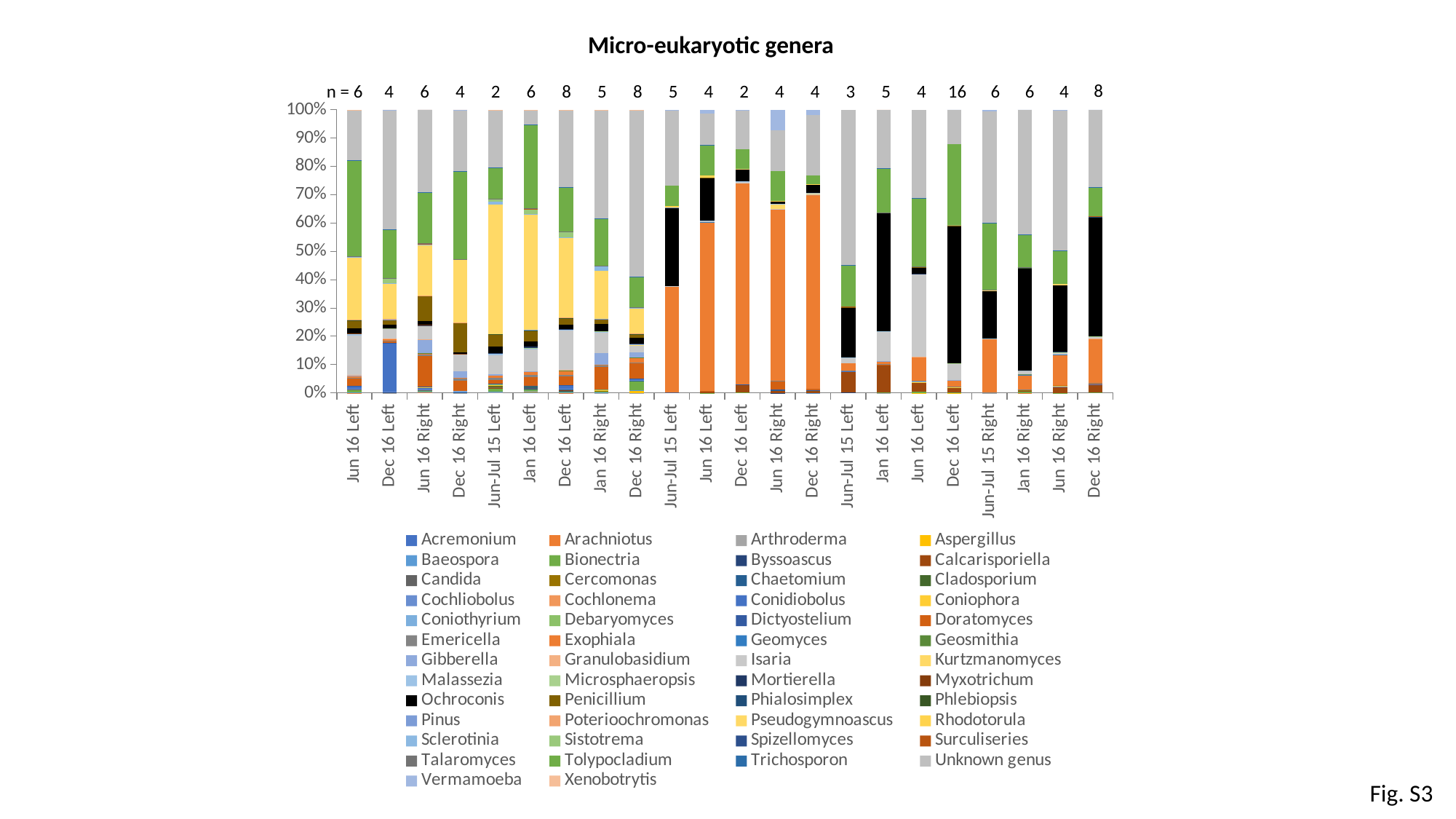

Micro-eukaryotic genera
8
n = 6
4
6
4
2
6
8
5
8
5
4
2
4
4
3
5
4
16
6
6
4
### Chart
| Category | Acremonium | Arachniotus | Arthroderma | Aspergillus | Baeospora | Bionectria | Byssoascus | Calcarisporiella | Candida | Cercomonas | Chaetomium | Cladosporium | Cochliobolus | Cochlonema | Conidiobolus | Coniophora | Coniothyrium | Debaryomyces | Dictyostelium | Doratomyces | Emericella | Exophiala | Geomyces | Geosmithia | Gibberella | Granulobasidium | Isaria | Kurtzmanomyces | Malassezia | Microsphaeropsis | Mortierella | Myxotrichum | Ochroconis | Penicillium | Phialosimplex | Phlebiopsis | Pinus | Poterioochromonas | Pseudogymnoascus | Rhodotorula | Sclerotinia | Sistotrema | Spizellomyces | Surculiseries | Talaromyces | Tolypocladium | Trichosporon | Unknown genus | Vermamoeba | Xenobotrytis |
|---|---|---|---|---|---|---|---|---|---|---|---|---|---|---|---|---|---|---|---|---|---|---|---|---|---|---|---|---|---|---|---|---|---|---|---|---|---|---|---|---|---|---|---|---|---|---|---|---|---|---|
| Jun 16 Left | 0.0 | 0.000624828172252631 | 0.00624828172252631 | 0.134962885206568 | 0.0618579890530104 | 0.940366399240209 | 0.0262427832346105 | 0.0306165804403789 | 0.0856014595986104 | 0.0537352228137262 | 0.0412386593686736 | 0.119967009072505 | 0.0349903776461473 | 0.00312414086126315 | 0.98098023043663 | 0.0212441578565894 | 0.0318662367848842 | 0.0124965634450526 | 0.0218689860288421 | 2.402464322311364 | 0.336157556671915 | 0.575466746644673 | 0.0 | 0.0 | 0.159331183924421 | 0.375521731523831 | 13.88368198745345 | 0.0 | 0.612331608807578 | 0.0393641748519157 | 0.0306165804403789 | 0.0106220789282947 | 1.665791907225513 | 2.997300742295869 | 0.0106220789282947 | 0.0187448451675789 | 0.00249931268901052 | 0.0612331608807578 | 21.95271300392391 | 0.0 | 0.481742520806778 | 0.0262427832346105 | 0.0 | 0.0787283497038314 | 0.0218689860288421 | 33.61138186998575 | 0.198695358776337 | 17.75136837369723 | 0.0612331608807578 | 0.0262427832346105 |
| Dec 16 Left | 0.0 | 0.0 | 0.0149958761340631 | 0.132151158431431 | 0.0 | 0.110594586488716 | 0.0140586338756842 | 0.0365524480767789 | 0.0459248706605683 | 0.0581090200194946 | 0.00562345355027367 | 0.0937242258378946 | 0.00656069580865262 | 0.00374896903351578 | 17.05312289120491 | 0.016870360650821 | 0.0 | 0.0112469071005473 | 0.0534228087275999 | 0.556721901477094 | 0.327097548174252 | 0.600772287620904 | 0.0 | 0.0 | 0.0740421384119367 | 0.0909124990627577 | 3.136012596535952 | 0.0 | 0.349591362375347 | 0.0131213916173052 | 0.000937242258378946 | 0.000937242258378946 | 1.237159781060209 | 1.749831296393491 | 0.0159331183924421 | 0.0384269325935368 | 0.0140586338756842 | 0.406763140136463 | 12.37909574866912 | 0.0 | 0.051548324210842 | 1.732023693484292 | 0.0 | 0.0534228087275999 | 0.0196820874259579 | 17.35866386743646 | 0.0215565719427157 | 42.0765539476644 | 0.0262427832346105 | 0.0121841493589263 |
| Jun 16 Right | 0.0 | 0.391767264002399 | 0.00624828172252631 | 0.0924745694933893 | 0.0224938142010947 | 0.428007297993052 | 0.0206193296843368 | 0.0306165804403789 | 0.0699807552922946 | 0.0624828172252631 | 0.0468621129189473 | 0.126840118967284 | 0.0356152058183999 | 0.0 | 0.732923446052336 | 0.0174951888230737 | 0.0106220789282947 | 0.0 | 0.00249931268901052 | 10.98447926820125 | 0.409887280997726 | 0.612331608807578 | 0.0 | 0.0312414086126315 | 4.825547974307065 | 0.0249931268901052 | 4.523755967109044 | 0.000624828172252631 | 0.228687111044463 | 0.0706055834645473 | 0.0681062707755367 | 0.132463572517558 | 1.369623353577766 | 9.0143960410887 | 0.0212441578565894 | 0.0156207043063158 | 0.0324910649571368 | 0.00624828172252631 | 17.64577241258653 | 0.0 | 0.431131438854315 | 0.016870360650821 | 0.146209792307116 | 0.0387393466796631 | 0.0349903776461473 | 17.99817550173702 | 0.0243682987178526 | 29.18010047237009 | 0.00124965634450526 | 0.0124965634450526 |
| Dec 16 Right | 0.0 | 0.0290545100097473 | 0.0140586338756842 | 0.0299917522681263 | 0.0131213916173052 | 0.0646697158281473 | 0.0159331183924421 | 0.034677963560021 | 0.0496738396940841 | 0.0309289945265052 | 0.0131213916173052 | 0.173389817800105 | 0.0140586338756842 | 0.0 | 0.248369198470421 | 0.0290545100097473 | 0.0187448451675789 | 0.0 | 0.00562345355027367 | 3.597135787658394 | 0.793844192846967 | 0.233373322336358 | 0.000937242258378946 | 0.0 | 2.229699332683512 | 0.0 | 5.974919397165778 | 0.0 | 0.112469071005473 | 0.0131213916173052 | 0.00656069580865262 | 0.0328034790432631 | 0.582027442453326 | 10.41744770188198 | 0.00562345355027367 | 0.00468621129189473 | 0.00468621129189473 | 0.136837369723326 | 22.31761265651945 | 0.000937242258378946 | 0.0496738396940841 | 0.00281172677513684 | 0.0 | 0.051548324210842 | 0.0121841493589263 | 31.10613331333883 | 0.0328034790432631 | 21.46659668591137 | 0.0140586338756842 | 0.0149958761340631 |
| Jun-Jul 15 Left | 0.0468621129189473 | 0.0 | 0.0281172677513684 | 0.307415460748294 | 0.103096648421684 | 1.124690710054735 | 0.164954637474695 | 0.0393641748519157 | 0.367398965284547 | 0.0299917522681263 | 0.0 | 0.506110819524631 | 0.0937242258378946 | 0.0 | 0.0 | 0.0468621129189473 | 0.221189172977431 | 0.0431131438854315 | 0.0224938142010947 | 1.482717252755492 | 0.618579890530104 | 0.796655919622104 | 0.0 | 0.0 | 0.521106695658694 | 0.0 | 6.845617455199821 | 0.0 | 0.322411336882357 | 0.331783759466147 | 0.00374896903351578 | 0.0 | 2.314988378195997 | 4.348804078878308 | 0.0 | 0.00374896903351578 | 0.0 | 0.0 | 45.72992427082552 | 0.0 | 1.105945864887156 | 0.946614680962735 | 0.0 | 0.0487365974357052 | 0.00749793806703157 | 10.88138261977956 | 0.136837369723326 | 20.3793956661918 | 0.0112469071005473 | 0.016870360650821 |
| Jan 16 Left | 0.161830496613431 | 0.0 | 0.196196046087326 | 0.176826372747494 | 0.0562345355027367 | 0.647321986453725 | 0.404263827447452 | 0.0212441578565894 | 0.149958761340631 | 0.0599835045362525 | 0.0718552398090525 | 0.366774137112294 | 0.0462372847466947 | 0.0 | 0.086226287770863 | 0.0537352228137262 | 0.0624828172252631 | 0.0124965634450526 | 0.00874759441153683 | 2.915448251730774 | 0.730424133363325 | 1.099072754992377 | 0.0 | 0.0 | 0.203693984154358 | 0.000624828172252631 | 7.870960485866387 | 0.0 | 0.208692609532379 | 0.131838744345305 | 0.621079203219115 | 0.0 | 1.858863812451576 | 3.885181575066857 | 0.0 | 0.0399890030241683 | 0.164329809302442 | 0.0 | 40.59633600759791 | 0.0 | 0.308665117092799 | 1.53895178825823 | 0.0 | 0.163080152957937 | 0.297418209992252 | 29.51188423183624 | 0.0837269750818525 | 5.109844792682012 | 0.00124965634450526 | 0.0762290370148209 |
| Dec 16 Left | 0.0131213916173052 | 0.00421759016270525 | 0.0370210692059684 | 0.0965359526130314 | 0.0164017395216316 | 0.388486916098073 | 0.356152058183999 | 0.0538914298567894 | 0.119029766814126 | 0.016870360650821 | 0.0384269325935368 | 0.220251930719052 | 0.0290545100097473 | 0.0 | 1.257779110744545 | 0.0398327959811052 | 0.0721676538951788 | 0.00984104371297893 | 0.0112469071005473 | 3.056347004573742 | 0.433005923371073 | 1.630801529579366 | 0.0 | 0.0262427832346105 | 0.243214366049336 | 0.0051548324210842 | 14.11533703231611 | 0.0 | 0.119967009072505 | 0.0941928469670841 | 0.0117155282297368 | 0.0 | 1.461160680812776 | 2.511809252455575 | 0.000468621129189473 | 0.025774162105421 | 0.052485566469221 | 0.160737047311989 | 28.18240608832571 | 0.00281172677513684 | 0.0927869835795156 | 1.877296243533028 | 0.0 | 0.0984104371297893 | 0.0510797030816525 | 15.52026317762615 | 0.0857576666416736 | 27.31405113593762 | 0.0121841493589263 | 0.0342093424308315 |
| Jan 16 Right | 0.0 | 0.0 | 0.0172452575541726 | 0.104221339131739 | 0.0179950513608758 | 0.399640098972782 | 0.0397390717552673 | 0.0292419584614231 | 0.116967833845692 | 0.0164954637474694 | 0.00149958761340631 | 0.19944515258304 | 0.0119967009072505 | 0.0 | 0.0517357726625178 | 0.0187448451675789 | 0.0734797930569094 | 0.0157456699407663 | 0.00899752568043788 | 8.126265277048812 | 0.355402264377296 | 0.317162780235435 | 0.0 | 0.0052485566469221 | 4.050386143810451 | 0.0 | 7.440203943915423 | 0.0 | 0.200194946389743 | 0.259428657119292 | 0.0112469071005473 | 0.0 | 2.428582139911524 | 1.796505960860764 | 0.0 | 0.0119967009072505 | 0.0329909274949389 | 0.0 | 16.95808652620529 | 0.0 | 1.778510909499888 | 0.000749793806703157 | 0.0 | 0.0419884531753768 | 0.0149958761340631 | 16.51720776786384 | 0.0742295868636125 | 38.41943465546974 | 0.000749793806703157 | 0.0352403089150484 |
| Dec 16 Right | 0.0 | 0.0 | 0.00234310564594736 | 0.821024218339957 | 0.0164017395216316 | 3.309402414336057 | 0.0393641748519157 | 0.0117155282297368 | 0.060452125665442 | 0.0154644972632526 | 0.00984104371297893 | 0.171515333283347 | 0.0140586338756842 | 0.0 | 0.605927120041988 | 0.0215565719427157 | 0.0473307340481368 | 0.000937242258378946 | 0.00562345355027367 | 5.37039814051136 | 0.263365074604484 | 1.708124015895628 | 0.0 | 0.00843518032541051 | 1.709061258154008 | 0.0 | 2.777048811576817 | 0.034677963560021 | 0.216034340556347 | 0.1068456174552 | 0.00234310564594736 | 0.0131213916173052 | 2.218921046712154 | 1.193109394916398 | 0.0243682987178526 | 0.0318662367848842 | 0.0581090200194946 | 0.103096648421684 | 8.86678038539402 | 0.000937242258378946 | 0.340687560920747 | 0.034677963560021 | 0.0 | 0.0496738396940841 | 0.00656069580865262 | 10.56272025193072 | 0.282578540901252 | 58.82272999925021 | 0.0112469071005473 | 0.0295231311389368 |
| Jun-Jul 15 Left | 0.0 | 0.0 | 0.0 | 0.0 | 0.0 | 0.0 | 0.0 | 0.0967234010647072 | 0.0 | 0.0 | 0.0 | 0.0 | 0.0 | 0.0 | 0.00449876284021894 | 0.0 | 0.00449876284021894 | 0.0 | 0.0194946389742821 | 0.000749793806703157 | 0.000749793806703157 | 37.4604483766964 | 0.194946389742821 | 0.0 | 0.0 | 0.0 | 0.0194946389742821 | 0.0 | 0.00299917522681263 | 0.0 | 0.00224938142010947 | 0.0 | 27.44620229436904 | 0.176951338381945 | 0.0 | 0.0 | 0.0314913398815326 | 0.0 | 0.00224938142010947 | 0.688310714553498 | 0.0 | 0.0 | 0.0 | 0.0 | 0.0 | 6.968583639499137 | 0.0 | 26.83287096048587 | 0.0464872160155957 | 0.0 |
| Jun 16 Left | 0.0 | 0.0 | 0.0 | 0.0 | 0.0 | 0.0196820874259579 | 0.0 | 0.525792906950589 | 0.0 | 0.0 | 0.0468621129189473 | 0.0140586338756842 | 0.00187448451675789 | 0.0 | 0.00843518032541051 | 0.0 | 0.0 | 0.0 | 0.0487365974357052 | 0.0253055409762315 | 0.00281172677513684 | 59.48957786608683 | 0.353340331408863 | 0.0 | 0.00187448451675789 | 0.0 | 0.0506110819524631 | 0.00374896903351578 | 0.254929894279073 | 0.0 | 0.00374896903351578 | 0.0 | 15.27517432706006 | 0.0393641748519157 | 0.0328034790432631 | 0.0459248706605683 | 0.0 | 0.0 | 0.0131213916173052 | 0.603584014396041 | 0.0 | 0.0 | 0.0 | 0.0 | 0.0 | 10.63582514808427 | 0.00187448451675789 | 11.18786083826948 | 1.313076403988903 | 0.0 |
| Dec 16 Left | 0.0 | 0.0 | 0.0 | 0.0 | 0.0 | 0.0487365974357052 | 0.0 | 2.73862187898328 | 0.0 | 0.0 | 0.0 | 0.00562345355027367 | 0.0 | 0.0 | 0.0 | 0.0 | 0.0 | 0.0 | 0.103096648421684 | 0.0 | 0.0 | 71.17417710129713 | 0.0 | 0.00187448451675789 | 0.0 | 0.0 | 0.654195096348504 | 0.0 | 0.0599835045362525 | 0.0 | 0.00187448451675789 | 0.0 | 4.228837069805802 | 0.0468621129189473 | 0.0243682987178526 | 0.0 | 0.0 | 0.0393641748519157 | 0.00562345355027367 | 0.0 | 0.00187448451675789 | 0.0 | 0.0 | 0.0 | 0.0 | 6.894354052635524 | 0.0 | 13.91804753692735 | 0.052485566469221 | 0.0 |
| Jun 16 Right | 0.0 | 0.0 | 0.0 | 0.0 | 0.0 | 0.0 | 0.00187448451675789 | 0.994414036140061 | 0.0 | 0.0 | 0.00749793806703157 | 0.0 | 0.0 | 0.214628477168779 | 0.0609207467946315 | 0.0 | 0.0 | 0.0 | 0.0309289945265052 | 3.030104221339132 | 0.0262427832346105 | 60.45306290770038 | 0.0 | 0.0 | 0.0 | 0.0 | 0.0656069580865262 | 1.817312738996776 | 0.0262427832346105 | 0.0 | 0.0 | 0.0 | 1.043150633575767 | 0.0684186848616631 | 0.0121841493589263 | 0.000937242258378946 | 0.0 | 0.0 | 0.00656069580865262 | 0.0740421384119367 | 0.0 | 0.0 | 0.0 | 0.0 | 0.0 | 10.32091174926895 | 0.0 | 14.6500337407213 | 7.094923895928619 | 0.0 |
| Dec 16 Right | 0.0 | 0.0 | 0.0 | 0.0 | 0.000937242258378946 | 0.000937242258378946 | 0.0 | 0.565157081802504 | 0.0 | 0.00187448451675789 | 0.0 | 0.00656069580865262 | 0.0 | 0.379583114643473 | 0.0224938142010947 | 0.0 | 0.0 | 0.0 | 0.0506110819524631 | 0.339281697533178 | 0.0112469071005473 | 68.38119517132787 | 0.0 | 0.0 | 0.00749793806703157 | 0.0 | 0.256804378795831 | 0.734797930569094 | 0.0206193296843368 | 0.0 | 0.0 | 0.0 | 2.758303966409238 | 0.0955987103546525 | 0.0599835045362525 | 0.0 | 0.00187448451675789 | 0.0955987103546525 | 0.00281172677513684 | 0.033740721301642 | 0.000937242258378946 | 0.0 | 0.0 | 0.0 | 0.0 | 3.037602159406163 | 0.0 | 21.24915648196746 | 1.88479418160006 | 0.0 |
| Jun-Jul 15 Left | 0.0 | 0.0 | 0.0 | 0.0 | 0.0 | 0.279923021169179 | 0.0199945015120842 | 7.259253705231063 | 0.0 | 0.0 | 0.0 | 0.033740721301642 | 0.0 | 0.0 | 0.164954637474695 | 0.0 | 0.0 | 0.0 | 0.0124965634450526 | 0.0362400339906526 | 0.0962235385269051 | 2.455574716952838 | 0.0 | 0.0 | 0.0 | 0.0 | 2.090675064357302 | 0.0 | 0.033740721301642 | 0.00374896903351578 | 0.0 | 0.0 | 17.70638074529504 | 0.182449826297768 | 0.0 | 0.0 | 0.0624828172252631 | 0.0 | 0.0387393466796631 | 0.0 | 0.00374896903351578 | 0.0 | 0.0 | 0.0124965634450526 | 0.00249931268901052 | 14.67096548449176 | 0.0774786933593262 | 54.75619204718703 | 0.0 | 0.0 |
| Jan 16 Left | 0.0 | 0.0 | 0.000749793806703157 | 0.00824773187373472 | 0.0 | 0.12821474094624 | 0.00149958761340631 | 9.806553197870585 | 0.0 | 0.00224938142010947 | 0.0 | 0.0329909274949389 | 0.000749793806703157 | 0.0 | 0.00299917522681263 | 0.0119967009072505 | 0.0 | 0.0 | 0.0 | 0.0494863912424083 | 0.0434880407887831 | 1.06170803029167 | 0.0 | 0.0 | 0.0149958761340631 | 0.0 | 10.32616030591587 | 0.0 | 0.247431956212042 | 0.00224938142010947 | 0.0 | 0.0 | 41.8309964759691 | 0.121466596685911 | 0.0 | 0.0052485566469221 | 0.0052485566469221 | 0.0 | 0.0269925770413136 | 0.0 | 0.00449876284021894 | 0.00374896903351578 | 0.0 | 0.0329909274949389 | 0.0254929894279073 | 15.45999850041239 | 0.103471545325036 | 20.63432556047087 | 0.0 | 0.00374896903351578 |
| Jun 16 Left | 0.0 | 0.0 | 0.0 | 0.00937242258378946 | 0.0 | 0.417072804978631 | 0.0 | 3.306590687560921 | 0.0 | 0.102159406163305 | 0.0 | 0.0140586338756842 | 0.0140586338756842 | 0.0 | 0.00749793806703157 | 0.00468621129189473 | 0.117155282297368 | 0.0 | 0.229624353302842 | 0.0712304116367999 | 0.0224938142010947 | 8.320836769888285 | 0.0 | 0.0 | 0.0 | 0.0431131438854315 | 29.39660343405563 | 0.0 | 0.0993476793881682 | 0.0 | 0.0206193296843368 | 0.0 | 2.054435030366649 | 0.283984404288821 | 0.0843518032541051 | 0.0 | 0.00562345355027367 | 0.0 | 0.016870360650821 | 0.0 | 0.0253055409762315 | 0.0 | 0.0 | 0.00374896903351578 | 0.00656069580865262 | 24.14710954487516 | 0.0159331183924421 | 31.15955612206644 | 0.0 | 0.0 |
| Dec 16 Left | 0.0 | 0.0 | 0.0 | 0.00866949089000525 | 0.0 | 0.061389367923821 | 0.00562345355027367 | 1.872844342805728 | 0.0 | 0.00796655919622104 | 0.00117155282297368 | 0.0513140136462473 | 0.00117155282297368 | 0.0379583114643473 | 0.000468621129189473 | 0.00913811201919472 | 0.0 | 0.0 | 0.00702931693784209 | 0.0562345355027367 | 0.0374896903351578 | 2.288042663267601 | 0.0 | 0.000234310564594737 | 0.0110125965359526 | 0.0 | 6.031153932668516 | 0.0 | 0.0475650446127315 | 0.0128870810527105 | 0.0 | 0.0 | 48.29749943765464 | 0.25328972032691 | 0.00562345355027367 | 0.000702931693784209 | 0.0 | 0.152536177551173 | 0.0545943615505736 | 0.000468621129189473 | 0.00398327959811052 | 0.00585776411486841 | 0.0 | 0.00609207467946315 | 0.000937242258378946 | 28.56948714103622 | 0.00679500637324736 | 12.09183099647597 | 0.000937242258378946 | 0.0 |
| Jun-Jul 15 Right | 0.0 | 0.0 | 0.0449876284021894 | 0.00124965634450526 | 0.0 | 0.0 | 0.0 | 0.0 | 0.0 | 0.0 | 0.0 | 0.00187448451675789 | 0.0 | 0.0 | 0.00374896903351578 | 0.0 | 0.0 | 0.0 | 0.0462372847466947 | 0.016870360650821 | 0.00937242258378946 | 18.94791432356102 | 0.0 | 0.0 | 0.00124965634450526 | 0.00499862537802104 | 0.00687310989477894 | 0.0 | 0.0312414086126315 | 0.00624828172252631 | 0.0 | 0.0 | 16.88660618329959 | 0.0824773187373472 | 0.0324910649571368 | 0.00124965634450526 | 0.00624828172252631 | 0.0 | 0.00874759441153683 | 0.386143810452126 | 0.0 | 0.0 | 0.0 | 0.00562345355027367 | 0.00249931268901052 | 23.44417785109095 | 0.0437379720576841 | 39.31606308265226 | 0.661068206243283 | 0.0 |
| Jan 16 Right | 0.0 | 0.000624828172252631 | 0.000624828172252631 | 0.00749793806703157 | 0.00249931268901052 | 0.358026542700757 | 0.00187448451675789 | 0.534852915448252 | 0.0174951888230737 | 0.139961510584589 | 0.00312414086126315 | 0.0293669240958736 | 0.00437379720576841 | 0.0 | 0.0487365974357052 | 0.00187448451675789 | 0.0 | 0.0318662367848842 | 0.0431131438854315 | 0.0393641748519157 | 0.0206193296843368 | 4.848666616680412 | 0.0949738821823998 | 0.329284446777136 | 0.00187448451675789 | 0.000624828172252631 | 1.115318287470945 | 0.0 | 0.103721476593937 | 0.00499862537802104 | 0.0 | 0.00312414086126315 | 36.11319387168528 | 0.144335307790358 | 0.0 | 0.00749793806703157 | 0.000624828172252631 | 0.0 | 0.263052660518357 | 0.00562345355027367 | 0.0 | 0.00124965634450526 | 0.0 | 0.00187448451675789 | 0.00187448451675789 | 11.64367298992777 | 0.00687310989477894 | 44.01414610981981 | 0.00749793806703157 | 0.0 |
| Jun 16 Right | 0.0 | 0.0 | 0.0 | 0.0 | 0.0 | 0.00124965634450526 | 0.0 | 2.259378670865512 | 0.0 | 0.0237434705456 | 0.0 | 0.0312414086126315 | 0.00874759441153683 | 0.0 | 0.0987228512159156 | 0.0187448451675789 | 0.0 | 0.0 | 0.183699482642273 | 0.0237434705456 | 0.00374896903351578 | 10.9032516058084 | 0.0662317862587788 | 0.0 | 0.00124965634450526 | 0.0 | 0.476119067256505 | 0.0 | 0.183699482642273 | 0.134962885206568 | 0.148709104996126 | 0.0 | 23.43980405388519 | 0.161205668441179 | 0.0 | 0.0 | 0.0 | 0.0 | 0.0262427832346105 | 0.178700857264252 | 0.00124965634450526 | 0.0 | 0.0 | 0.0 | 0.0 | 11.88423183624503 | 0.00749793806703157 | 49.64009897278248 | 0.0937242258378946 | 0.0 |
| Dec 16 Right | 0.0 | 0.0 | 0.0 | 0.0562345355027367 | 0.0 | 0.0234310564594736 | 0.00234310564594736 | 2.895609957261753 | 0.00140586338756842 | 0.0328034790432631 | 0.0 | 0.00656069580865262 | 0.0 | 0.0 | 0.110125965359526 | 0.00609207467946315 | 0.0449876284021894 | 0.0 | 0.0403014171102947 | 0.116686661168179 | 0.100284921646547 | 15.77284996625928 | 0.0262427832346105 | 0.00140586338756842 | 0.00656069580865262 | 0.00328034790432631 | 0.529541875984105 | 0.0 | 0.156050836020094 | 0.0164017395216316 | 0.0 | 0.0 | 42.14872160155957 | 0.147615655694684 | 0.0210879508135263 | 0.00562345355027367 | 0.000937242258378946 | 0.0295231311389368 | 0.0913811201919472 | 0.0243682987178526 | 0.000468621129189473 | 0.0 | 0.0 | 0.0262427832346105 | 0.00234310564594736 | 10.23890305166079 | 0.1246532203644 | 27.17861962960185 | 0.0103096648421684 | 0.0 |Fig. S3

## Slide 7
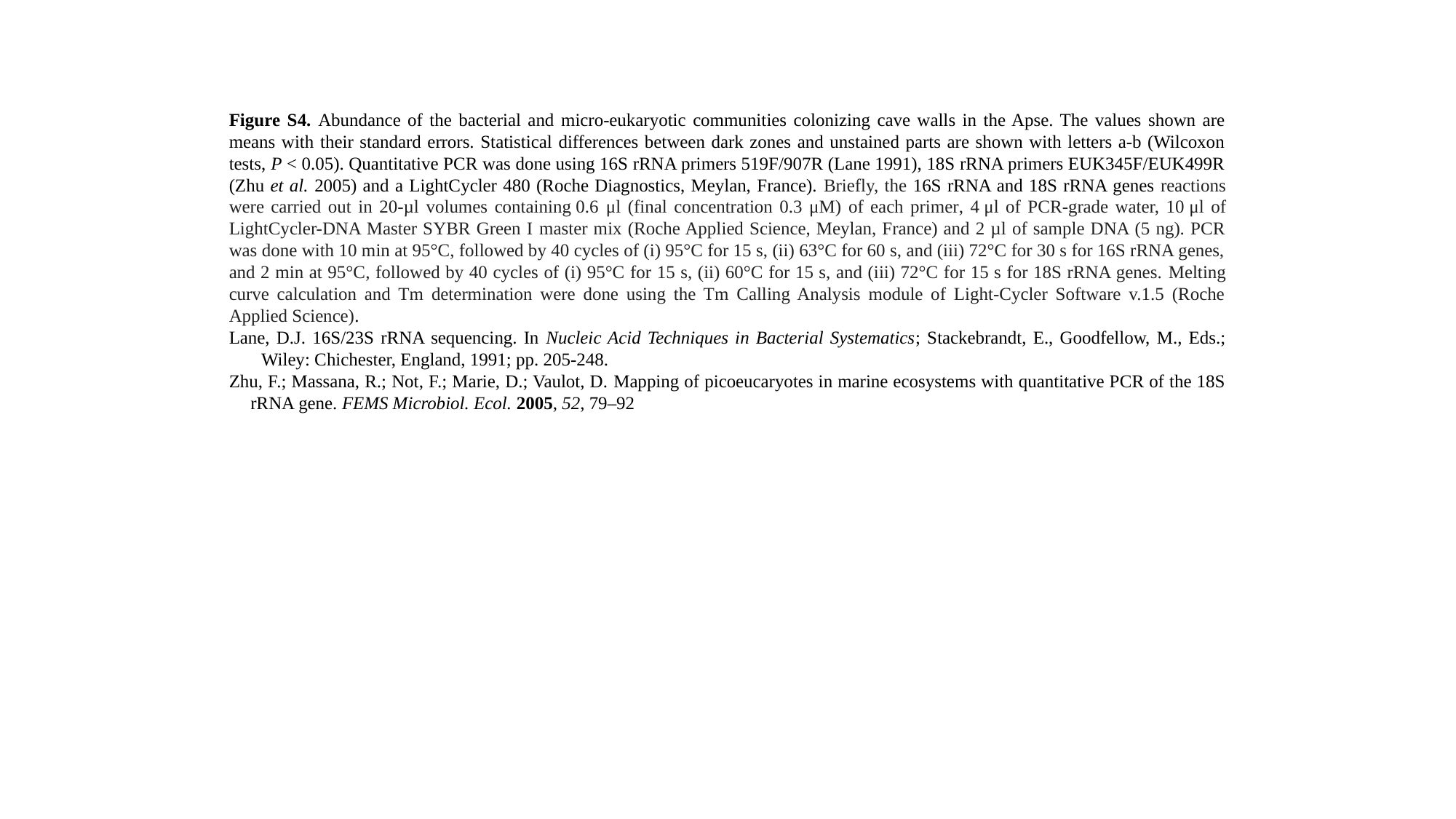

Figure S4. Abundance of the bacterial and micro-eukaryotic communities colonizing cave walls in the Apse. The values shown are means with their standard errors. Statistical differences between dark zones and unstained parts are shown with letters a-b (Wilcoxon tests, P < 0.05). Quantitative PCR was done using 16S rRNA primers 519F/907R (Lane 1991), 18S rRNA primers EUK345F/EUK499R (Zhu et al. 2005) and a LightCycler 480 (Roche Diagnostics, Meylan, France). Briefly, the 16S rRNA and 18S rRNA genes reactions were carried out in 20-µl volumes containing 0.6 μl (final concentration 0.3 μM) of each primer, 4 μl of PCR-grade water, 10 μl of LightCycler-DNA Master SYBR Green I master mix (Roche Applied Science, Meylan, France) and 2 µl of sample DNA (5 ng). PCR was done with 10 min at 95°C, followed by 40 cycles of (i) 95°C for 15 s, (ii) 63°C for 60 s, and (iii) 72°C for 30 s for 16S rRNA genes, and 2 min at 95°C, followed by 40 cycles of (i) 95°C for 15 s, (ii) 60°C for 15 s, and (iii) 72°C for 15 s for 18S rRNA genes. Melting curve calculation and Tm determination were done using the Tm Calling Analysis module of Light-Cycler Software v.1.5 (Roche Applied Science).
Lane, D.J. 16S/23S rRNA sequencing. In Nucleic Acid Techniques in Bacterial Systematics; Stackebrandt, E., Goodfellow, M., Eds.; Wiley: Chichester, England, 1991; pp. 205-248.
Zhu, F.; Massana, R.; Not, F.; Marie, D.; Vaulot, D. Mapping of picoeucaryotes in marine ecosystems with quantitative PCR of the 18S rRNA gene. FEMS Microbiol. Ecol. 2005, 52, 79–92

## Slide 8
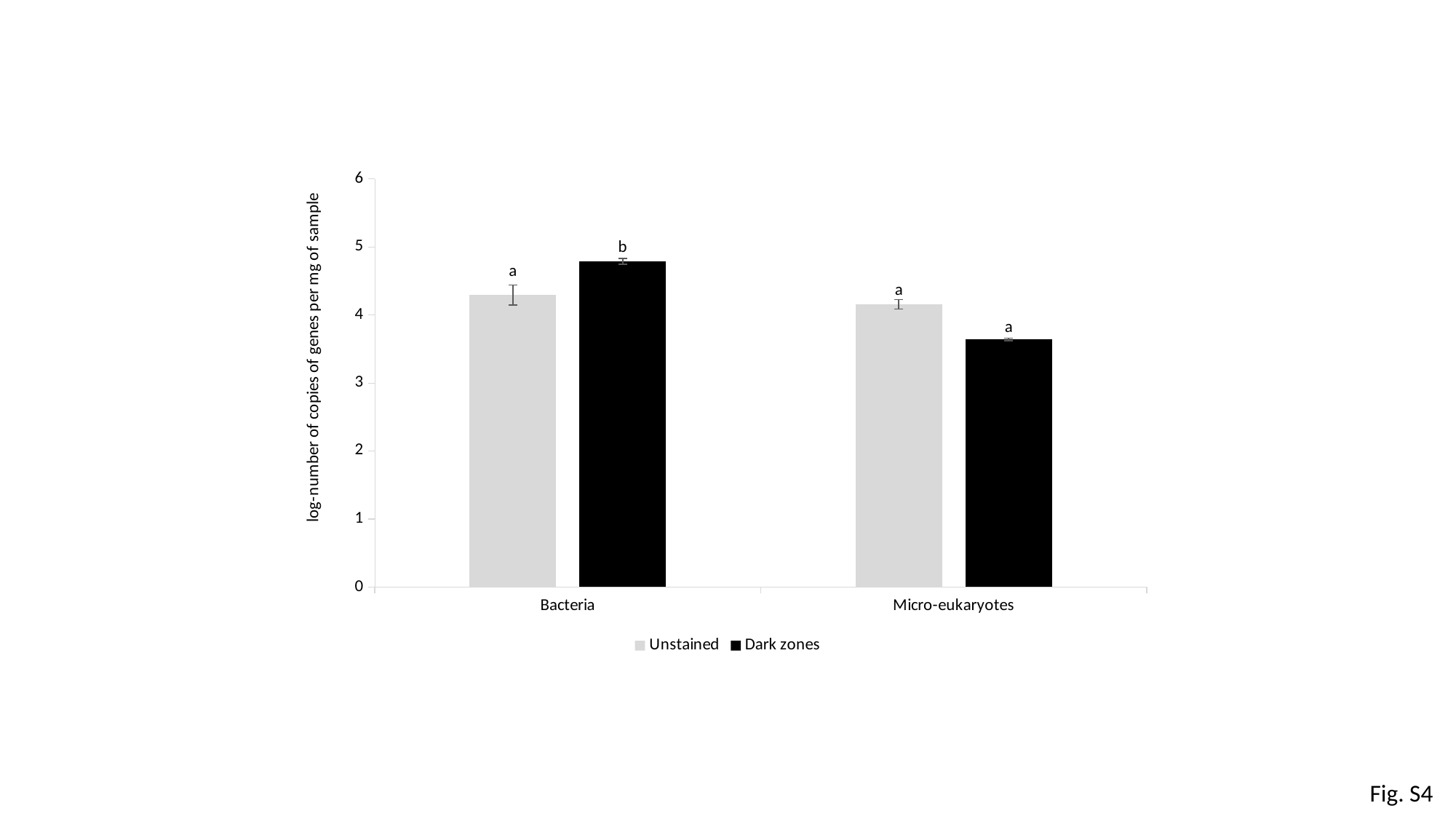

### Chart
| Category | Unstained | Dark zones |
|---|---|---|
| Bacteria | 4.29330156845602 | 4.785911644908271 |
| Micro-eukaryotes | 4.156884462123975 | 3.641069838961107 |Fig. S4

## Slide 9
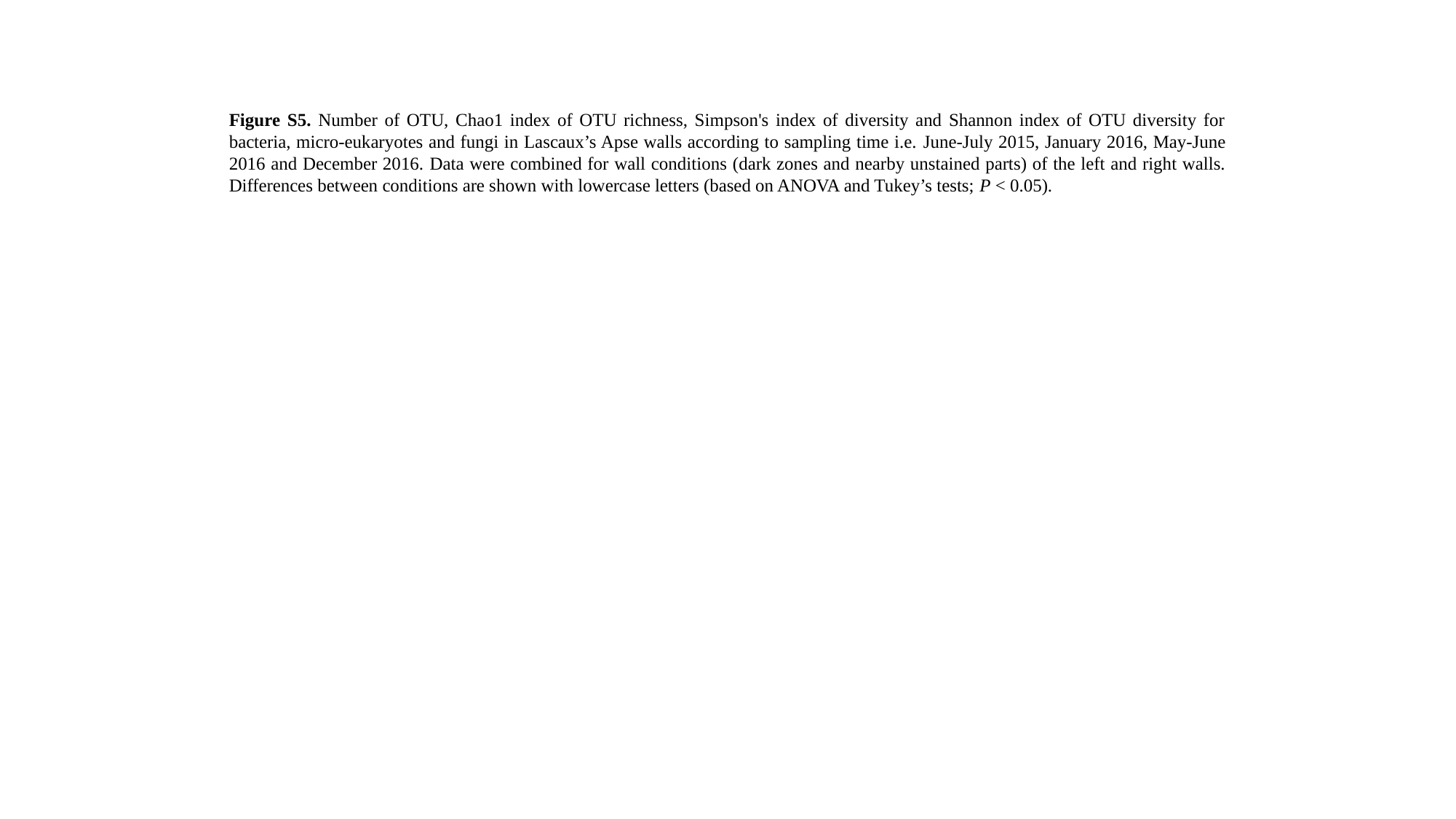

Figure S5. Number of OTU, Chao1 index of OTU richness, Simpson's index of diversity and Shannon index of OTU diversity for bacteria, micro-eukaryotes and fungi in Lascaux’s Apse walls according to sampling time i.e. June-July 2015, January 2016, May-June 2016 and December 2016. Data were combined for wall conditions (dark zones and nearby unstained parts) of the left and right walls. Differences between conditions are shown with lowercase letters (based on ANOVA and Tukey’s tests; P < 0.05).

## Slide 10
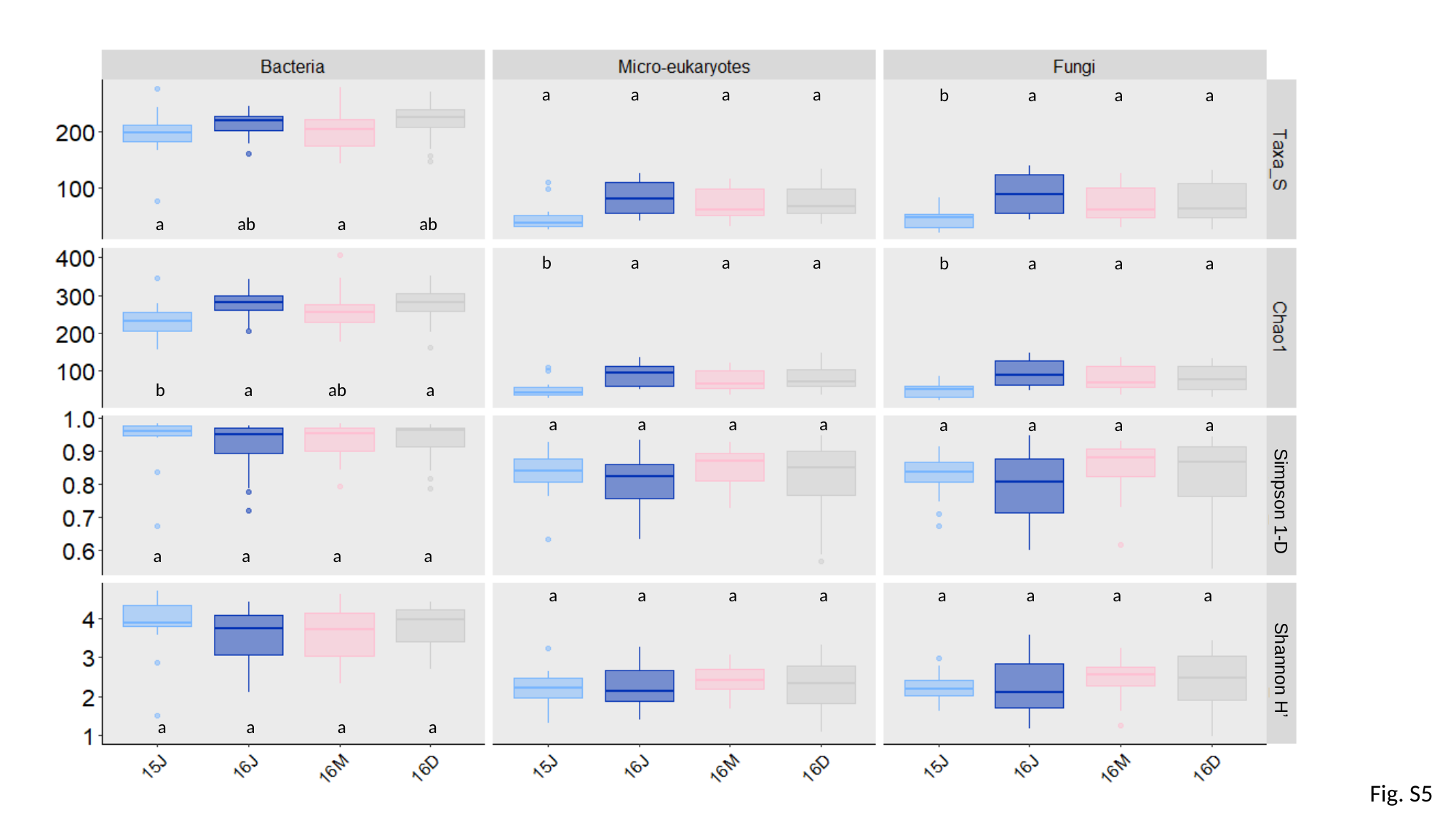

a
a
a
a
b
a
a
a
a
ab
a
ab
b
a
a
a
b
a
a
a
b
a
ab
a
a
a
a
a
a
a
a
a
a
a
a
a
a
a
a
a
a
a
a
a
a
a
a
a
 Simpson 1-D
 Shannon H’
Fig. S5

## Slide 11
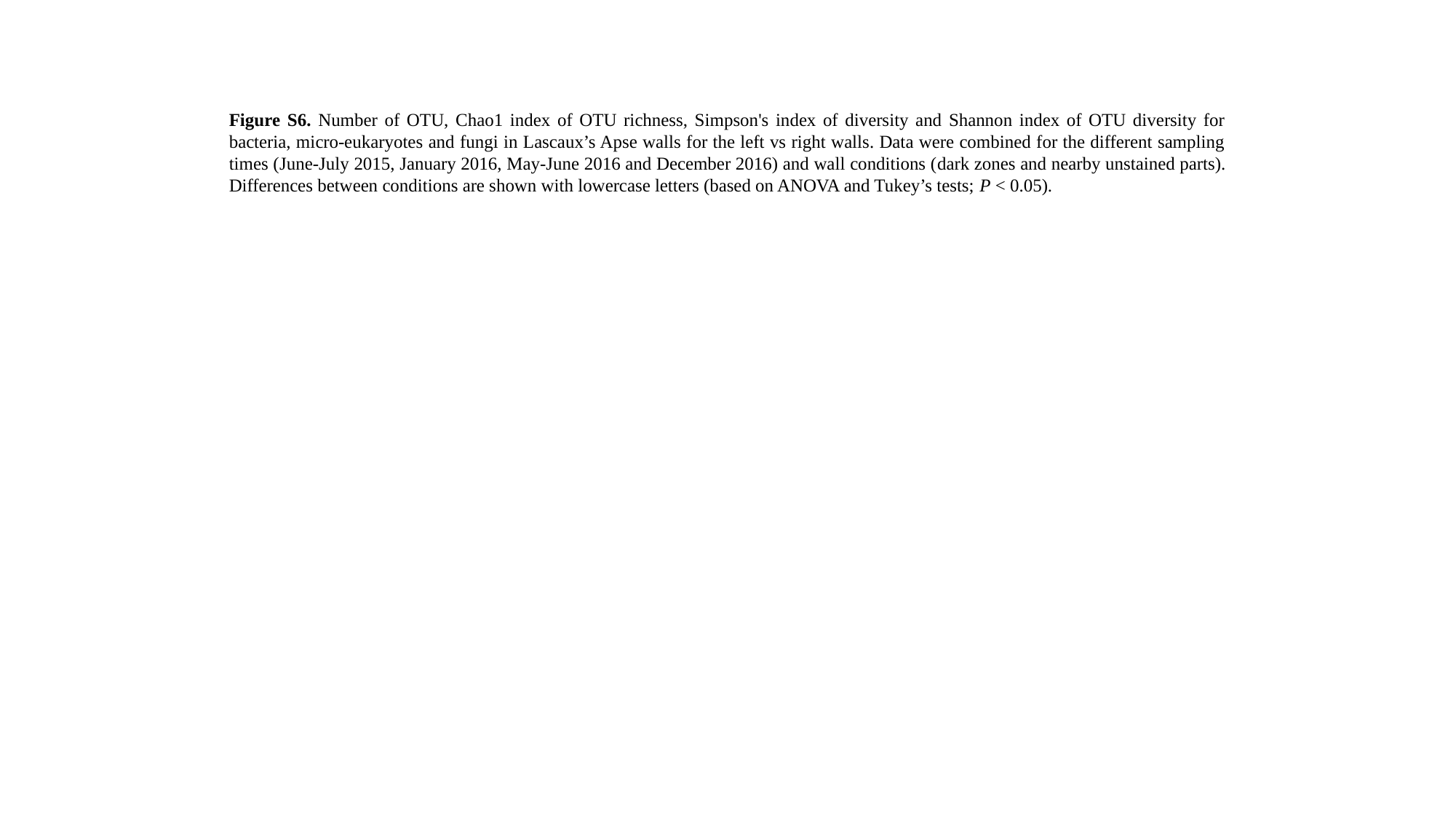

Figure S6. Number of OTU, Chao1 index of OTU richness, Simpson's index of diversity and Shannon index of OTU diversity for bacteria, micro-eukaryotes and fungi in Lascaux’s Apse walls for the left vs right walls. Data were combined for the different sampling times (June-July 2015, January 2016, May-June 2016 and December 2016) and wall conditions (dark zones and nearby unstained parts). Differences between conditions are shown with lowercase letters (based on ANOVA and Tukey’s tests; P < 0.05).

## Slide 12
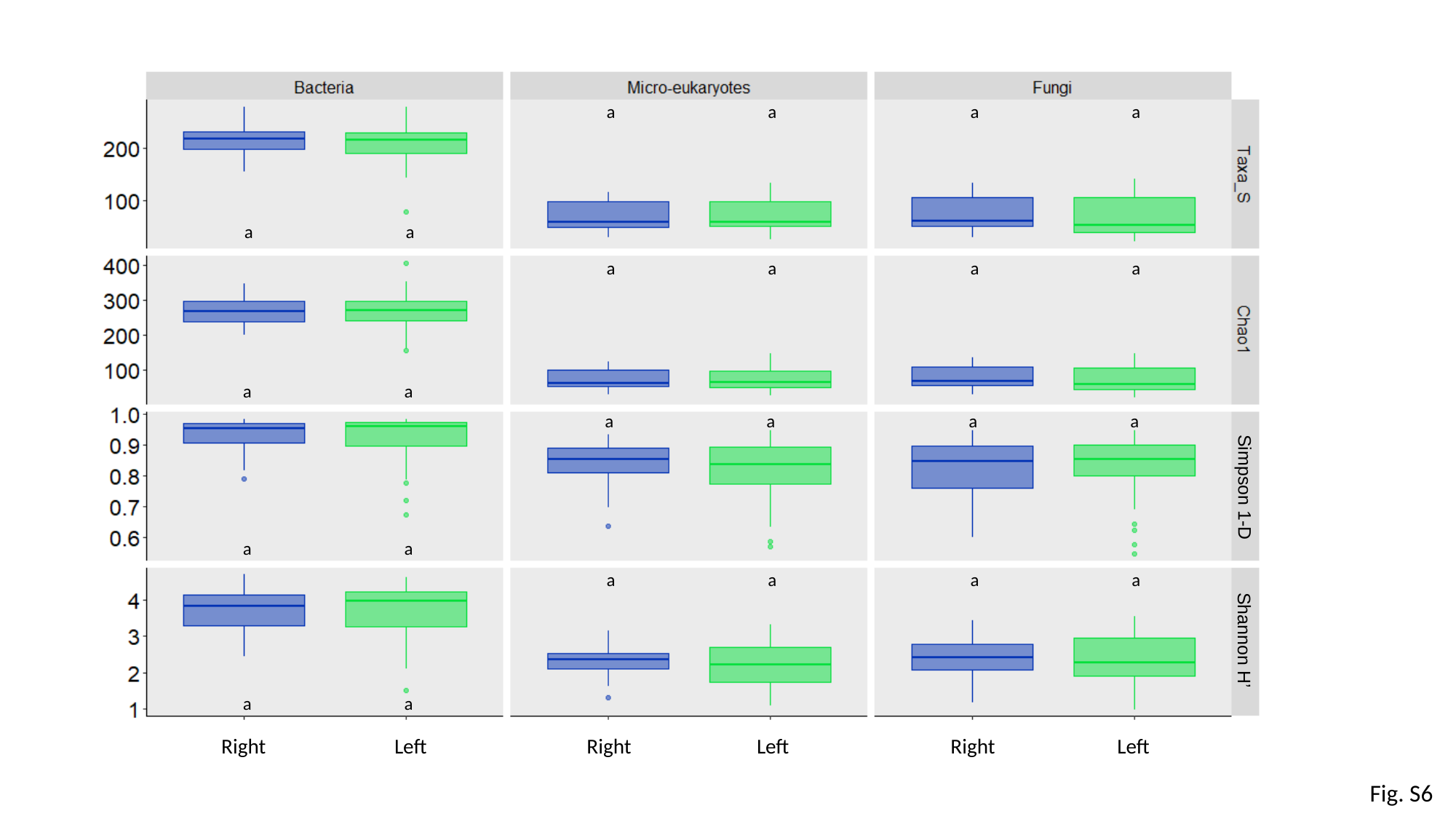

a
a
a
a
a
a
a
a
a
a
a
a
a
a
a
a
a
a
a
a
a
a
a
a
 Simpson 1-D
 Shannon H’
Right
Left
Right
Left
Right
Left
Fig. S6

## Slide 13
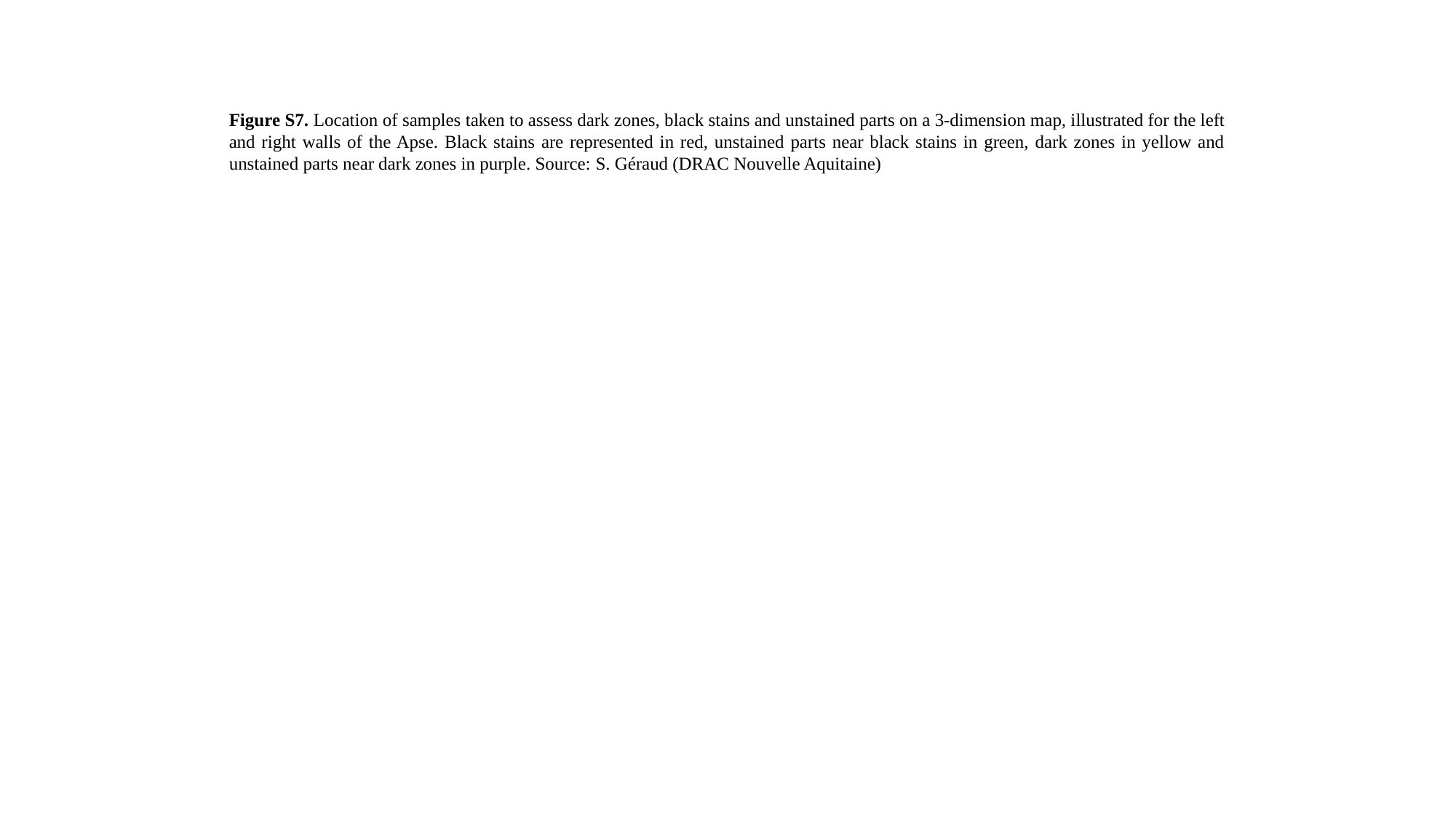

Figure S7. Location of samples taken to assess dark zones, black stains and unstained parts on a 3-dimension map, illustrated for the left and right walls of the Apse. Black stains are represented in red, unstained parts near black stains in green, dark zones in yellow and unstained parts near dark zones in purple. Source: S. Géraud (DRAC Nouvelle Aquitaine)

## Slide 14
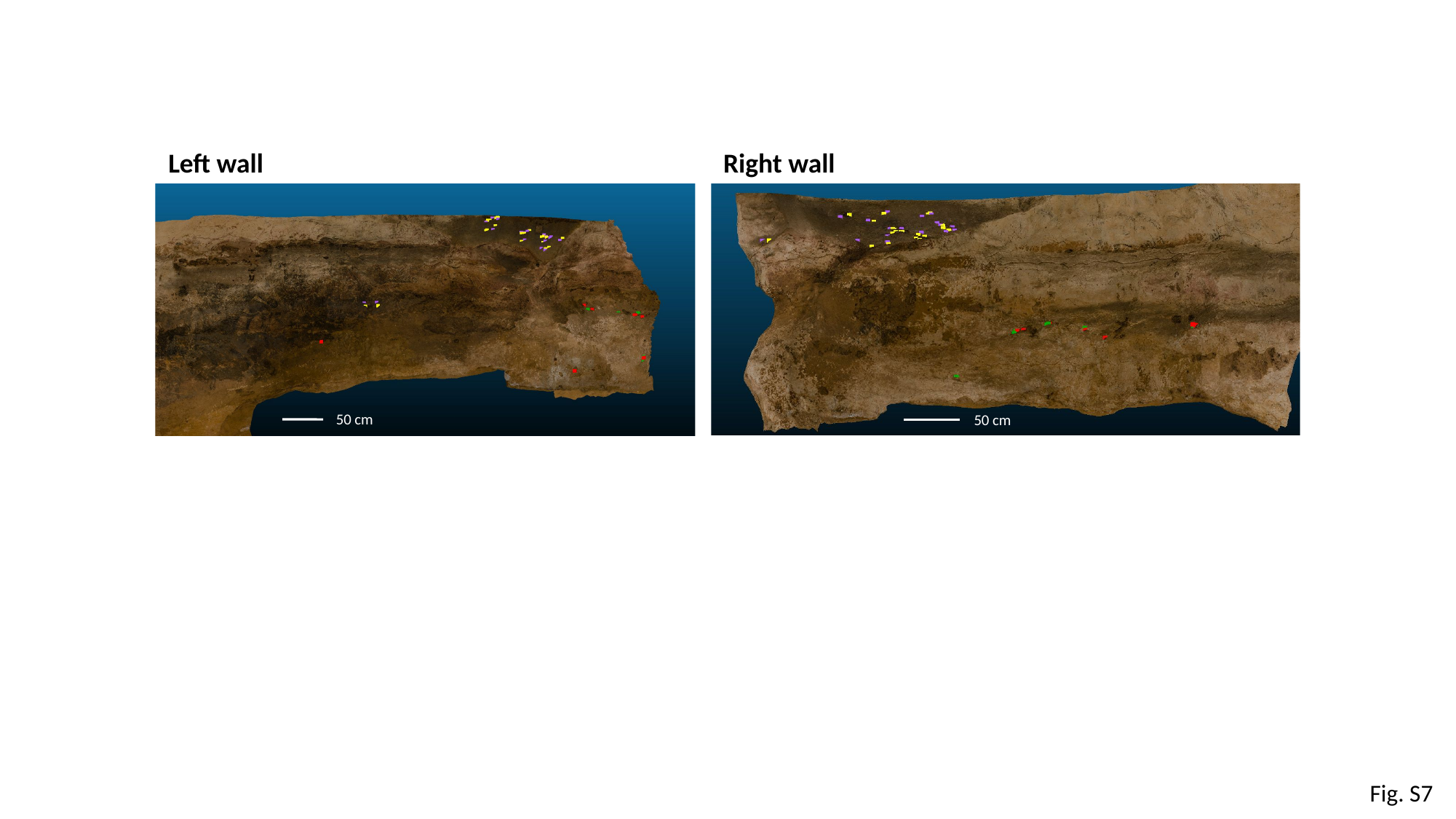

Left wall
Right wall
50 cm
50 cm
Fig. S7

## Slide 15
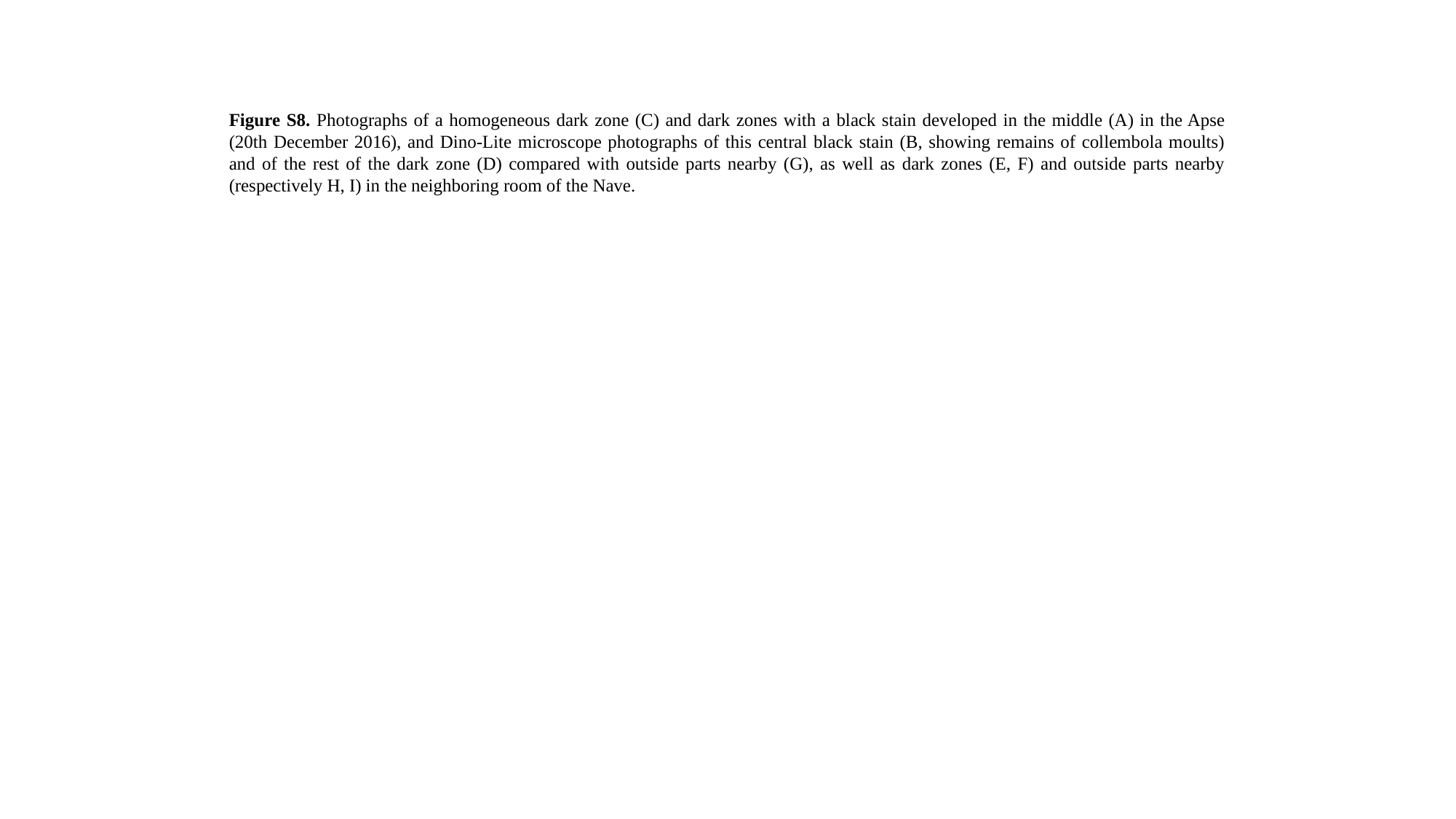

Figure S8. Photographs of a homogeneous dark zone (C) and dark zones with a black stain developed in the middle (A) in the Apse (20th December 2016), and Dino-Lite microscope photographs of this central black stain (B, showing remains of collembola moults) and of the rest of the dark zone (D) compared with outside parts nearby (G), as well as dark zones (E, F) and outside parts nearby (respectively H, I) in the neighboring room of the Nave.

## Slide 16
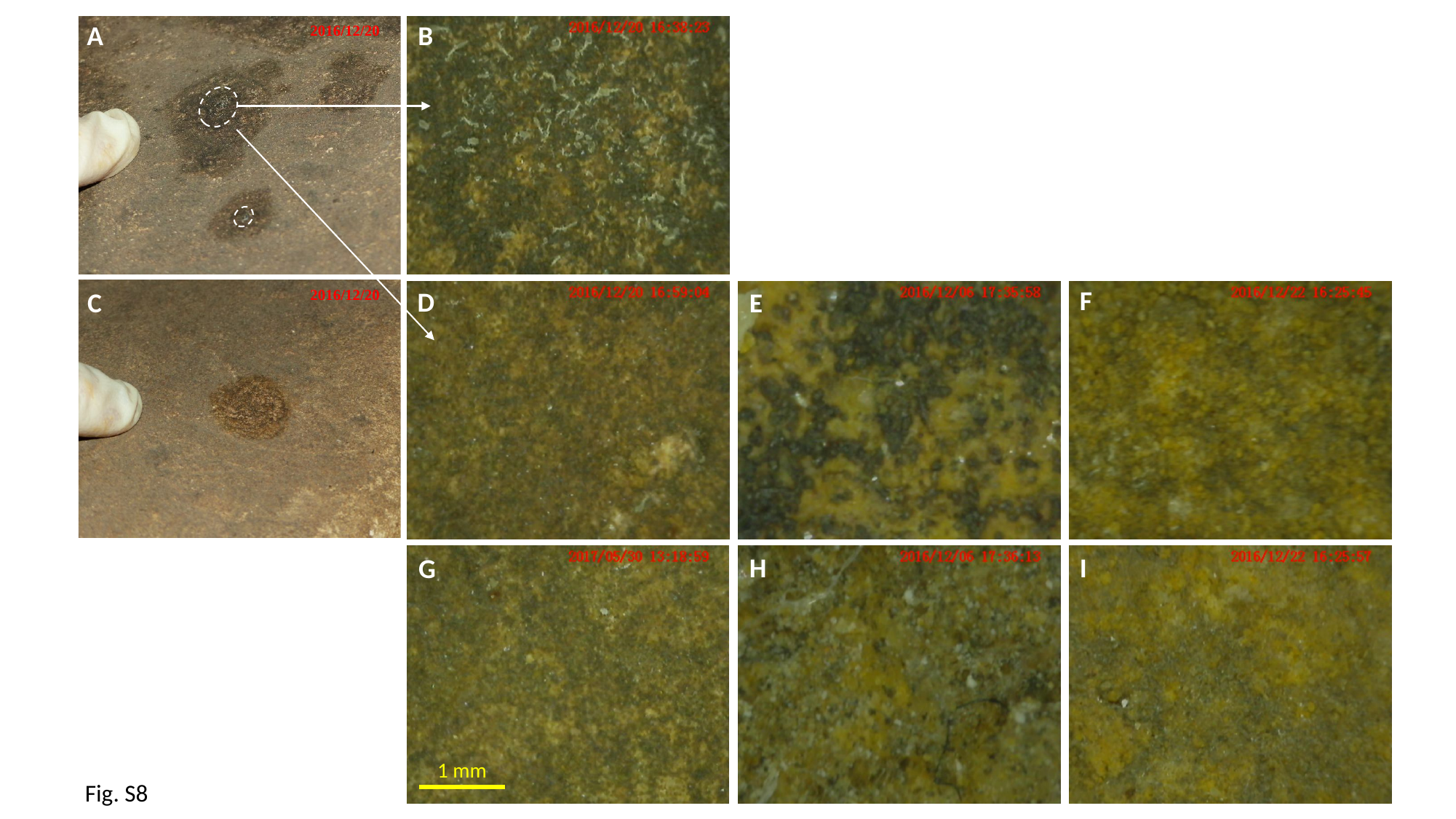

B
A
2016/12/20
F
2016/12/20
D
E
C
I
H
G
1 mm
Fig. S8

## Slide 17
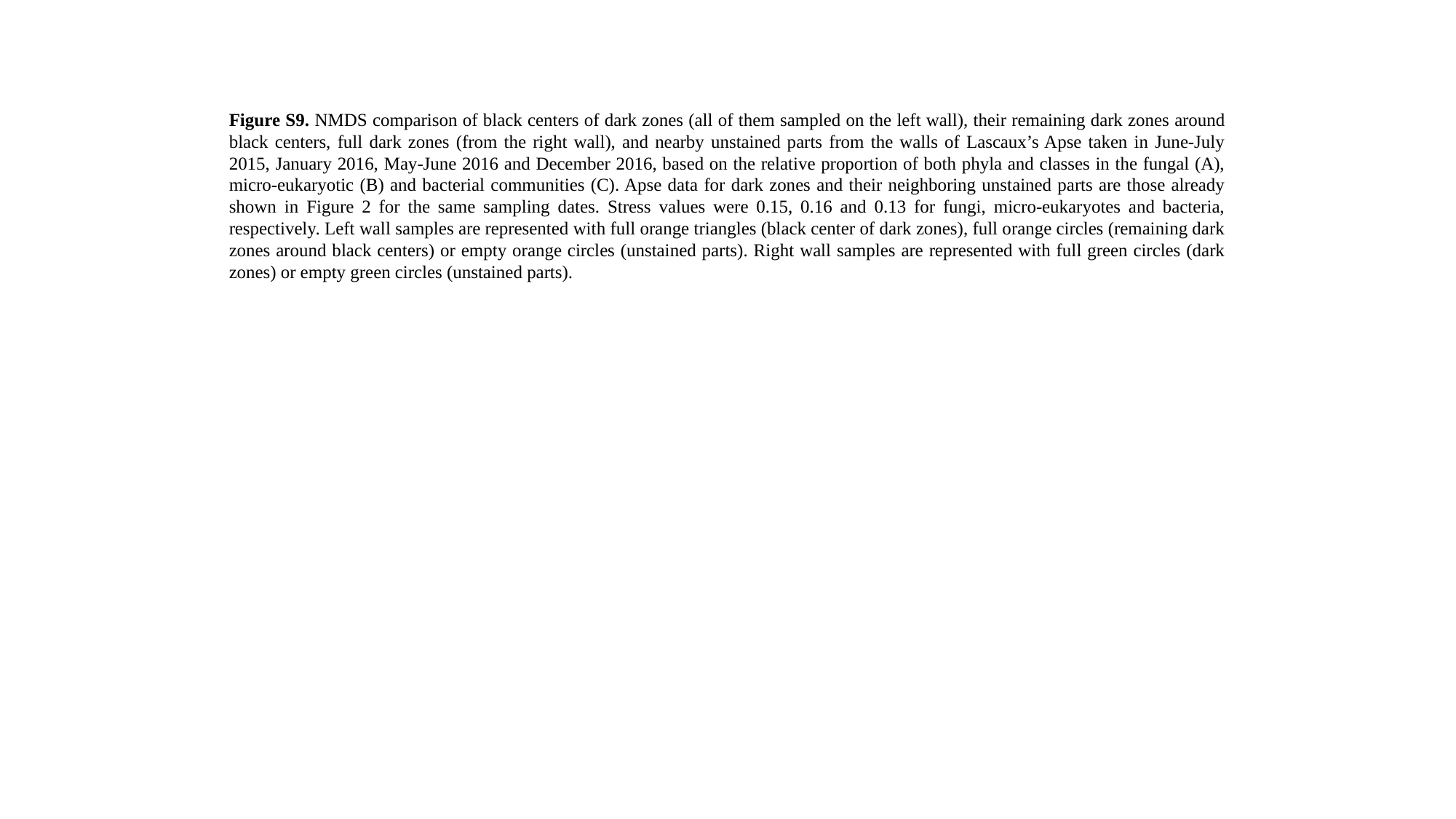

Figure S9. NMDS comparison of black centers of dark zones (all of them sampled on the left wall), their remaining dark zones around black centers, full dark zones (from the right wall), and nearby unstained parts from the walls of Lascaux’s Apse taken in June-July 2015, January 2016, May-June 2016 and December 2016, based on the relative proportion of both phyla and classes in the fungal (A), micro-eukaryotic (B) and bacterial communities (C). Apse data for dark zones and their neighboring unstained parts are those already shown in Figure 2 for the same sampling dates. Stress values were 0.15, 0.16 and 0.13 for fungi, micro-eukaryotes and bacteria, respectively. Left wall samples are represented with full orange triangles (black center of dark zones), full orange circles (remaining dark zones around black centers) or empty orange circles (unstained parts). Right wall samples are represented with full green circles (dark zones) or empty green circles (unstained parts).

## Slide 18
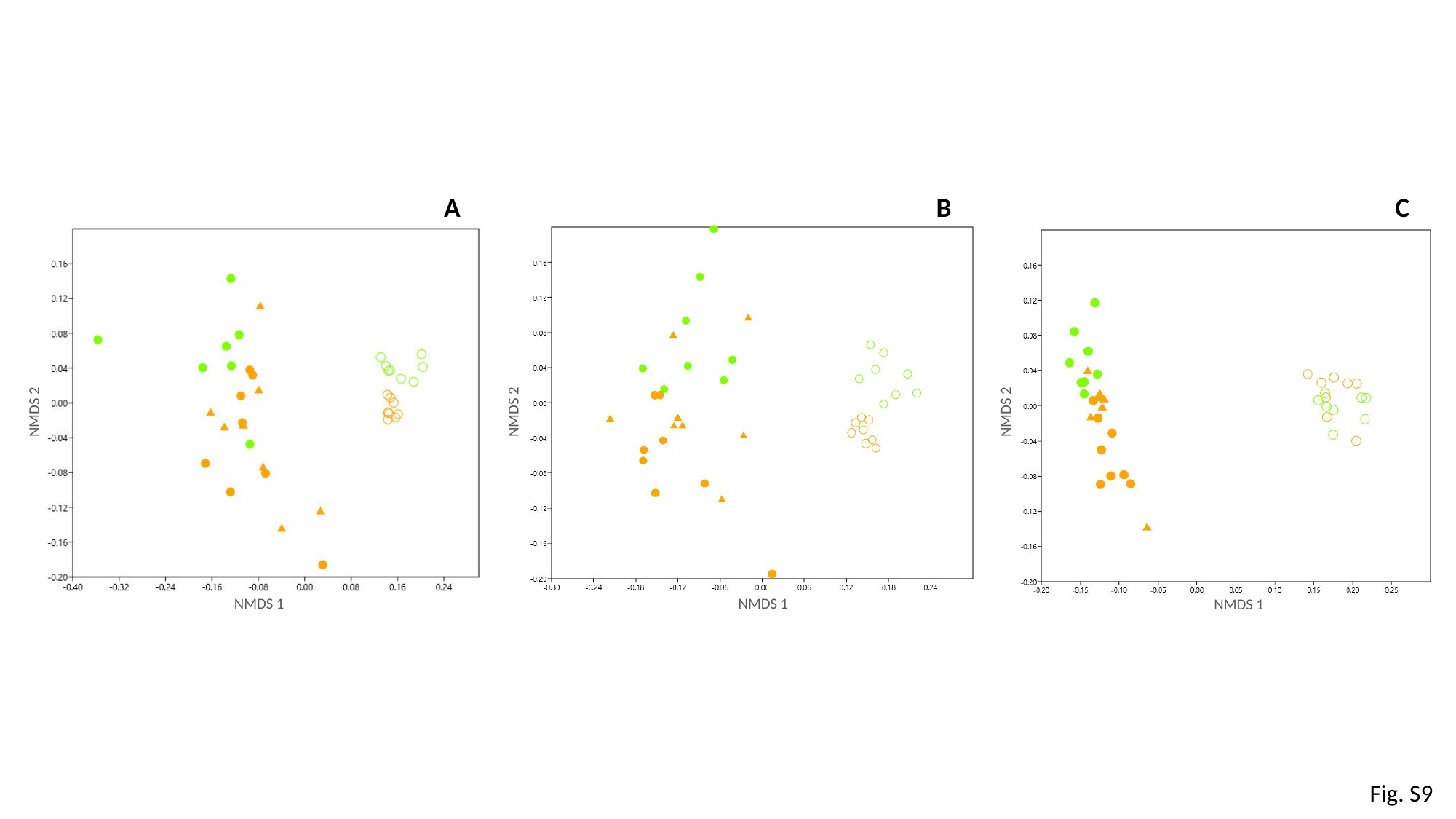

A
B
C
NMDS 2
NMDS 2
NMDS 1
NMDS 1
NMDS 1
NMDS 2
Fig. S9
